# Beyond Family: Modeling Non-hereditary Heart Diseases With Human Pluripotent Stem Cell-Derived Cardiomyocytes

**DOI:** 10.3389/fphys.2020.00384

**Published:** 2020-04-22

**Authors:** Sebastian Martewicz, Michael Magnussen, Nicola Elvassore

**Affiliations:** ^1^Shanghai Institute for Advanced Immunochemical Studies (SIAIS), ShanghaiTech University, Shanghai, China; ^2^Stem Cells & Regenerative Medicine Section, UCL Great Ormond Street Institute of Child Health, London, United Kingdom; ^3^Venetian Institute of Molecular Medicine, Padua, Italy; ^4^Department of Industrial Engineering, University of Padova, Padua, Italy

**Keywords:** ischemia – reperfusion, diabetes, non-genetic diseases, HPSC-cardiomyocytes, hPSC-CM, maladaptive hypertrophy

## Abstract

Non-genetic cardiac pathologies develop as an aftermath of extracellular stress-conditions. Nevertheless, the response to pathological stimuli depends deeply on intracellular factors such as physiological state and complex genetic backgrounds. Without a thorough characterization of their *in vitro* phenotype, modeling of maladaptive hypertrophy, ischemia and reperfusion injury or diabetes in human pluripotent stem cell-derived cardiomyocytes (hPSC-CMs) has been more challenging than hereditary diseases with defined molecular causes. In past years, greater insights into hPSC-CM *in vitro* physiology and advancements in technological solutions and culture protocols have generated cell types displaying stress-responsive phenotypes reminiscent of *in vivo* pathological events, unlocking their application as a reductionist model of human cardiomyocytes, if not the adult human myocardium. Here, we provide an overview of the available literature of pathology models for cardiac non-genetic conditions employing healthy (or asymptomatic) hPSC-CMs. In terms of numbers of published articles, these models are significantly lagging behind monogenic diseases, which misrepresents the incidence of heart disease causes in the human population.

## Introduction on hPSC-CMS

Nearly two decades since their first description (Kehat et al., 2001), hPSC-CMs are beginning to fulfill their potential as a reductionist model of the human cardiac muscle. Thanks to constant improvements in differentiation protocols (Mummery et al., 2003; Laflamme et al., 2007; Yang et al., 2008; Kattman et al., 2011; Lian et al., 2012; Burridge et al., 2014) and increasing understanding of their *in vitro* cardiac phenotype, hPSC-CMs are now an integral part of proposed high-throughput drug screening (Kirby et al., 2018; Fiedler et al., 2019) and drug risk-assessment platforms (Yang and Papoian, 2018; Lu H.R. et al., 2019; Li et al., 2020). Furthermore, there is evidence for their increasing reliability in predicting adverse drug effects (Blinova et al., 2018).

**FIGURE 1:**
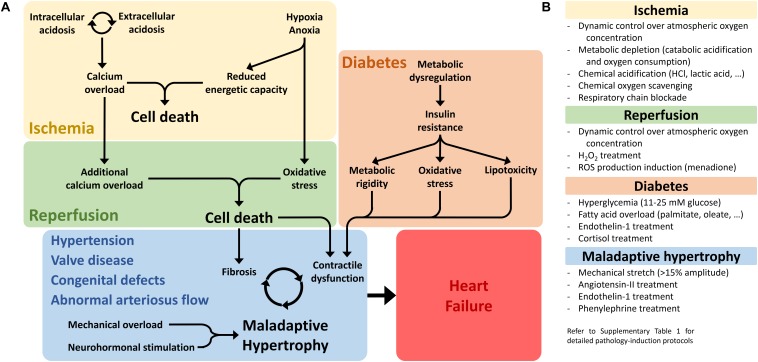
Non-genetic pathological conditions leading to heart failure. **(A)** The three main pathological conditions discussed in this review are schematically represented, highlighting the major molecular drivers and pathological phenotypes that need to be reproduced *in vitro* in order to generate a representative and reliable pathology model. **(B)** Main experimental strategies employed to generate pathological phenotypes in non-genetic cardiac disease models *in vitro*. For detailed experimental protocols employed in the reviewed studies, see Supplementary Materials.

The successful induction of pluripotency in human somatic cells (Takahashi et al., 2007; Yu et al., 2007; Lowry et al., 2008; Park et al., 2008) opened the cardiac field to patient-specific disease modeling (Carvajal-Vergara et al., 2010; Moretti et al., 2010), although patient-specific treatment modeling still remains an open challenge (Blinova et al., 2019). The race toward generating mutation-specific *in vitro* models produced >150 independent hiPSC lines over the past 10 years and hundreds of scientific papers frequently and comprehensively reviewed (Ross et al., 2018; van Mil et al., 2018; van den Brink et al., 2019). Consequently, there is a clear literature unbalance against non-genetic cardiac pathology models, often coming with additional challenges in recreating *in vitro* either the pathological phenotype, the pathological environment or both (Figure 1).

Here, we discuss modeling of non-genetic heart conditions, focusing exclusively on results obtained on human cells when the referenced study makes only sparing use of hPSC-CMs

## Advantages and Limitations

Inter-species differences are a major concern in translational research. Therefore, the human origin paired with virtually unlimited low-cost supply constitute the most valuable advantages of hPSC-CMs. Beyond the most often quoted heart size, beating rate, electrophysiology and protein function (Nerbonne et al., 2001; Haghighi et al., 2003), more subtle differences are apparent also in stress-responses. For instance, an *in vitro* angiotensin-II-induced heart failure model reproduces the appearance observed in failing myocardia of two loss-of-function Na_*V*_1.5 channel isoforms produced by abnormal *SCN5A* splicing through a mechanism absent in species other than primates (Gao et al., 2011, 2013). Such response contributes to the sodium current reduction in angiotensin-II-treated hPSC-CMs, mimicking pro-arrhythmic conditions in failing ventricles (Mathieu et al., 2016). Similarly, evolutionarily closer species display divergent transcriptomic responses to ischemia-mimetic environments, with rhesus macaque monkey PSC-CMs failing to overlap results with hPSC-CMs at gene regulation level (Zhao et al., 2018), and chimpanzee PSC-CMs still diverging in regulation of critical genes tightly related to human ischemia/reperfusion pathogenesis (Ward and Gilad, 2019).

Although hPSC-CMs can develop full adult phenotypes, these have been achieved so far only by integration within healthy animal myocardia (Cho et al., 2017; Kadota et al., 2017), and hPSC-CM developmental immaturity is seen as their major drawback. We (Martewicz et al., 2019) and others (van den Berg et al., 2015) have shown that transcriptomic profiling places hPSC-CMs within the first trimester of fetal development, with structural, functional and metabolic features further supporting such characterization (Machiraju and Greenway, 2019).

Nevertheless, unprimed hPSC-CMs (no maturation protocol applied) still represent a valid reductionist model in dissecting molecular mechanisms within human and cardiac cell backgrounds. For instance, a recent study successfully identified direct inactivation mechanisms of human voltage-sensitive L-type calcium channels by molecular O_2_ and acidosis (Fernandez-Morales et al., 2019), complementing our findings in murine models (Martewicz et al., 2012). Simultaneously, the authors clearly show how studying more complex functional features requires careful evaluation of cardiac structural maturation, with whole-cell ion dynamics changing following substrate interaction, which our group showed to be mediated by mechanotransduction signaling (Martewicz et al., 2017).

Additionally, taking advantage of developmentally early phenotypes of hPSC-CM and hijacking the differentiation process from hPSCs allows modeling developmental defects leading to postnatal pathological conditions. Such is the case of hypoplastic left heart syndrome in a chronic-hypoxia model (Gaber et al., 2013), which preceded patient-specific hPSC-CMs models ultimately identifying the underlying genetic-driven molecular mechanisms (Jiang et al., 2014; Kobayashi et al., 2014; Tomita-Mitchell et al., 2016; Hrstka et al., 2017; Yang et al., 2017). Similarly, hPSC-CMs were used to model the role of the mitochondrial calcium uniporter in cardiac fetal development and maturation (Shanmughapriya et al., 2018). Finally, although chemically induced cardiotoxicity will not be a subject of this review [see (Magdy et al., 2018)], one recent study considered the impact of ethanol on hPSC-CM functionality as a model of prenatal exposure during maternal alcohol intoxication (Rampoldi et al., 2019).

## Maladaptive Hypertrophy Modeling

The developmentally early phenotype of hPSC-CMs provides additional complexity in modeling hypertrophy *in vitro*, with differentiation/maturation phenomena blurring distinctions between physiological and pathological hypertrophy. Physiological hypertrophic growth is a cardiac perinatal maturation process, reactivated in adulthood upon regular physical activity, and differs substantially from pathological (or maladaptive) hypertrophy in activation mechanisms and elicited functional responses (McMullen and Jennings, 2007). For instance, the evaluation of cell-size increase must be performed carefully, being an ambivalent hallmark for both processes (Rupert et al., 2017), and appears to be absent in advanced maturation stages (Ronaldson-Bouchard et al., 2018). Similarly ambivalent is the application of mechanical stretch, which simultaneously induces hypertrophic responses and promotes hPSC-CM maturation (LaBarge et al., 2019), generating phenotypes divergent from pathological neurohormonal stimulation relative to αMHC/βMHC transcription activation ratios (Foldes et al., 2011) or CathepsinD/TroponinT release (Hoes et al., 2019).

Chronic adrenergic activation is one of the pathogenic triggers of maladaptive hypertrophy, and the effects of prolonged exposure to isoproterenol or phenylephrine have been studied in hPSC-CMs in regard to hypertrophy-inhibiting effects of several active compounds (Foldes et al., 2011; Martin et al., 2014; Gesmundo et al., 2017). Nevertheless, the reliability of this approach is hindered by hPSC-CM immature adrenergic signaling (Jung et al., 2016; Uzun et al., 2016; Trieschmann et al., 2019), which generates highly variable and aberrant stress-responses (Foldes et al., 2014) often failing to produce representative pathological phenotypes *in vitro* (Tanaka et al., 2014; Cui et al., 2016; Naftali-Shani et al., 2018).

Hormonal stimulation has been shown to be more effective for maladaptive hypertrophy modeling purposes, with angiotensin-II and especially endothelin-1 treatments successfully recapitulating hypertrophic phenotypes in terms of expression/secretion of natriuretic peptides A and B (Carlson et al., 2013), myofibrillar disarray (Tanaka et al., 2014) and mRNA/miRNA profiling (Aggarwal et al., 2014). Such a model has been dually employed thus far to study the molecular mechanisms of maladaptation *in vitro* (Cui et al., 2016; Rosales and Lizcano, 2018), and evaluate anti-hypertrophic effects of miRNAs (Scrimgeour et al., 2019), herbal extracts (Zhang et al., 2017) and antiparasitic compounds (Qin et al., 2017), for which hPSC-CMs are superior to murine cardiac cell lines lacking in expression of several key target proteins (Nagai et al., 2017).

Alternatively to being employed as *in vitro* hypertrophy modeling platform, hPSC-CMs have proven useful in experimentally confirming observations made in human and murine hypertrophic heart biopsies of the involvement of non-coding RNAs in maladaptive pathogenesis (Wang et al., 2016; Mirtschink et al., 2019).

## Ischemia/Reperfusion Injury Modeling

Ischemia is the most dramatic of cardiac insults, leading to or aggravating pre-existing stages of heart failure. The nature of the pathological stressors (a composite of fast dynamic changes in nutrients, waste products, O_2_ and ROS) makes cellular responses and pathological fallouts tightly connected to adult cardiomyocyte metabolic processes, elevating hPSC-CM maturation to a necessity for modeling purposes.

Indeed, several studies describe little or no response to I/R-mimetic conditions in unprimed hPSC-CMs, although showing minimal but relatively significant cardioprotection by the individual molecules of interest (Hsieh et al., 2015; Wei et al., 2017; Mo et al., 2019). Our own experiments with oxygen/glucose deprivation in microfluidic devices show clearly divergent responses of postnatal murine cardiomyocytes and unprimed hPSC-CMs characterized by abnormal intracellular glycogen stores (Martewicz et al., 2018). Similar experimental setups have been used to study the mechanistic action of anesthetics (Lu Y. et al., 2019) and miRNA-based regulation of metalloproteases (Scrimgeour et al., 2019).

Recent studies demonstrate how developing a stress-responsive phenotype must be set as an essential element in a feasible hPSC-CM model for I/R studies. Priming hPSC-CMs through simple maturation steps generates cells responsive to I/R with mortality rates unseen in their unprimed counterparts, ultimately providing the biological model needed to test clinically effective small molecules (Hidalgo et al., 2018) or investigate the cardioprotective mechanism of cardiac progenitors (Sebastiao et al., 2019). The most intriguing example of *in vitro* I/R modeling to date fully embraces hPSC-CMs as platform for both drug screening and development (Fiedler et al., 2019). The researchers identify MAP4K4 as a druggable target, activity of which is altered across several clinically relevant heart failure models, and employ an I/R setup with primed hPSC-CMs to screen for suitable small-molecule inhibitors. After using the identified lead-compound to develop a *novel* inhibitor, they ultimately translate the cardioprotective properties of a small-molecule newly developed in hPSC-CMs to an *in vivo* murine model of ischemic insult.

## Diabetes Modeling

Similar to ischemia models, replicating diabetic pathophysiology *in vitro* requires primed hPSC-CMs as starting point. While underlying genetic factors might further its severity, prolonged exposure to altered metabolic stimuli is the leading trigger and driving force of the clinical manifestations of diabetic cardiomyopathy (Graneli et al., 2019). Indeed, an I/R model that linked anesthetic-conferred cardioprotection to pharmacological tuning of mitochondrial function in hPSC-CMs (Sepac et al., 2010; Canfield et al., 2016) produced no differences between diabetic patient-specific cells and healthy controls. Both showed equal abrogation of protection under acute hyperglycemic conditions, thus failing to replicate the clinical differences between healthy and diabetic surgery patients (Canfield et al., 2012).

On the other hand, when allowed to adapt to prolonged exposure to hyperglycemic stress, hPSC-CMs develop pathological hypertrophy characterized by contractile and calcium cycling dysfunctions (Ng et al., 2018). Capitalizing on this phenotype has enabled investigations into mechanisms behind unexpected clinical trial evidence of empagliflozin-driven reduction of deadly cardiovascular complications in diabetic patients. Similar approaches of metabolic overload with fatty acids allow the induction of insulin-resistance and dissection of its mechanism in hPSC-CMs (Chanda et al., 2017; Liu et al., 2017; Graneli et al., 2019).

Primed hPSC-CMs develop a complete panel of diabetic cardiomyopathy phenotypes by integrating metabolic overload conditions with additional hormonal stimulation abnormally present in the diabetic milieu (Idris-Khodja et al., 2016; Joseph and Golden, 2017), proven by aggravated contractile dysfunction following endothelin-1 stimulation (Wu et al., 2018). A complete set of stressors (metabolic overload, endothelin-1 and cortisol treatment) recapitulates *in vitro* hypertrophic-like transcriptomic changes, increased BNP secretion, compromised calcium cycling and contraction, lipid accumulation and oxidation, sarcomeric disorganization (Drawnel et al., 2014), insulin-resistance and reduced respiratory capacity (Graneli et al., 2019), and deregulated non-coding RNAs expression (Pant et al., 2019). Satisfying all of these conditions in such a multifactorial pathological setting provides the necessary platform for drug-screening experiments and is instrumental in revealing underlying differences between healthy and patient-derived hPSC-CMs (Drawnel et al., 2014).

## Other Pathology Models

Hypertrophy, ischemia/reperfusion and diabetes are conditions with major economic and social impacts. Nevertheless, hPSC-CMs have been also employed in modeling less common pathological settings, such as systemic pathogen infections leading to myocarditis and heart failure. Modeling septic shock by exposure to bacterial lipopolysaccharides affects hPSC-CM survival, electrophysiology and demonstrates their competence in activating innate immune inflammatory responses (Yucel et al., 2017). Indeed, hPSC-CM display stronger macrophage chemo-attractant properties than purified chemokines (Pallotta et al., 2015) and significant stress-responsive paracrine pro-inflammatory signaling (Sebastiao et al., 2020) mediating fibrosis *in vivo* and *in vitro* (Kumar et al., 2019; Zhang et al., 2019). Furthermore, functional expression of coxsackievirus and adenovirus receptor (Scassa et al., 2011) makes hPSC-CMs a better predictive model than murine cardiac cell lines for therapeutic approaches against viral myocarditis (Sharma et al., 2014). Similarly, hPSC-CMs are a viable host for parasites causing Chagas disease (da Silva Lara et al., 2018; Bozzi et al., 2019) and, consequently, a good screening platform for novel drugs preventing infection and major cardiac fallouts of the pathology (Sass et al., 2019a, b).

Spaceflight-associated stressors such as radiation and microgravity induce cardiac atrophy and arrhythmias, increasing cardiovascular complication rates in astronauts (Acharya et al., 2019). Thus far, the intrinsic challenges of hPSC-CM aerospace applications limit *in vitro* models to phenotypic descriptions, orphan of underlying molecular mechanisms. Microgravity modeling, for instance, has been performed only twice on human PSC-CMs, observing increases in beating rate under acute conditions during parabolic flight (Acharya et al., 2019) and mainly transcriptomic changes during chronic exposure onboard the International Space Station (Wnorowski et al., 2019). Although studied relative to anti-cancer treatment, radiation-induced heart disease is another astronaut concern, and hPSC-CMs respond to ionizing radiations in dose-dependent manner with electrophysiological (Becker et al., 2018b) and transcriptomic (Becker et al., 2018a) alterations.

## Disease Modeling With 3D Constructs

hPSC-CM maturation *in vitro* is relatively fast in comparison with *in vivo* development, supporting the idea that these mechanisms differ substantially. Thus, modeling non-genetic pathologies mostly originating from insults to the adult heart in the late stages of cardiac development is within reach of 1,2 month-long cultures. While originally proposed as a maturation mechanism (Sartiani et al., 2007; Otsuji et al., 2010; Kamakura et al., 2013; Lundy et al., 2013), extended time in culture was recently extensively characterized over a 4-month period showing expression of aging markers in unprimed hPSC-CMs and increased sensitivity to I/R in disorganized 3D aggregates (Acun et al., 2019). To date, human engineered heart tissues (hEHTs) provide the closest match to an adult cardiomyocyte phenotype *in vitro* (Tiburcy et al., 2017; Ronaldson-Bouchard et al., 2018).

hEHTs assemble hPSC-CMs into 3D constructs integrating multifactorial stimuli such as electrophysiological pacing (Lemme et al., 2019; Zhao et al., 2019), mechanical loading (Leonard et al., 2018), ECM structure (Goldfracht et al., 2019) and non-myocyte cell interactions (Varzideh et al., 2019). Such constructs, in their immature state, have been proposed as models to study human cardiac self-regenerative potential after localized injury (Voges et al., 2017), and produce structurally and metabolically primed hPSC-CMs when allowed to develop further, even in absence of additional stimuli (Ulmer et al., 2018).

A recent hEHT I/R model showed for the first time in human cells the cardioprotective effect of ischemic preconditioning and efficacy of one out of three proposed cardioprotection treatments for reperfusion injury (Chen and Vunjak-Novakovic, 2019). Nevertheless, similar hEHTs generated by pure hPSC-CM populations are limited in their maturation potential (Park et al., 2019) and limit the study of the complex bidirectional crosstalk of multiple cell types, important during ischemic stress *via* paracrine signaling (Sandstedt et al., 2018; Sebastiao et al., 2019, 2020) and neurohormonal stimulation. The latter can be effectively modeled solely with hEHTs, as 2D cultures lack or display functionally impaired a β-adrenergic signaling cascades (Jung et al., 2016; Uzun et al., 2016; Trieschmann et al., 2019), despite being able to form functional sympathetic neuro-junctions (Sakai et al., 2017). Indeed, chronic exposure of hEHTs to norepinephrine induces contractile dysfunction and β-adrenergic desensitization, which with additional endothelin-1-driven hypertrophic stimulation, generates an advanced model of heart failure (Tiburcy et al., 2017). Notably, endothelin -1 treatment does not produce additional hypertrophic growth in hPSC-CM at such late stages of maturation, but induces more clinically relevant hypertrophic features, such as contractile dysfunction (Ronaldson-Bouchard et al., 2018). Additionally, porcine scaffold-based hEHTs have been employed recently to highlight the vicious cycle of maladaptive hypertrophy, with healthy hPSC-CMs responding to hypertrophic ECMs with impaired function, which *in vivo* would feed-back to the cardiac microenvironment triggering additional maladaptation preventing recovery under pharmacological treatment (Sewanan et al., 2019).

## Conclusion and Future Perspectives

**FIGURE 2:**
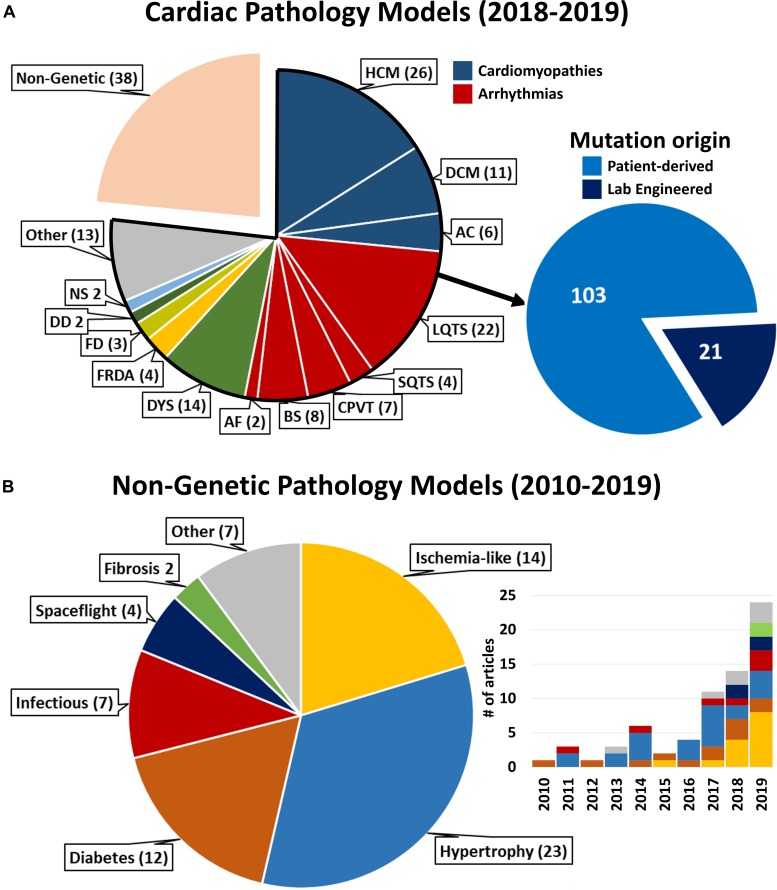
Literature statistics on cardiac pathology models and cited references. **(A)** Complete representation of pathological models for cardiac diseases available on PubMed between January 1st 2018 and January 2nd 2020. HCM, Hypertrophic Cardiomyopathy; DCM, Dilated Cardiomyopathy; AC, Arrhythmogenic Cardiomyopathy; L/SQTS, Long/Short QT Syndrome; CPVT, Catecholaminergic Polymorphic Ventricular Tachycardia; BS, Brugada Syndrome; AF, Atrial Fibrillation; DYS, Muscular Dystrophies [Duchenne (9), Myotonic (4), Limb-Girdle (1)]; FRDA, Friedriech’s Ataxia; FD, Fabry Disease; DD, Danon Disease; NS, Noonan Syndrome; Other, different genetic diseases represented by (1) article. *Inset*: all cardiac genetic pathology models reported in 2018–2019 relative to cell model derivation. “Lab engineered”: healthy hPSC genetically edited to carry the mutation under consideration. **(B)** Representation of non-genetic conditions referenced in this review according to the pathological condition modeled. *Inset*: distribution *per* year of publication of the referenced articles (same color coding). For bibliographical research methods and full list of references reported in this figure, see Supplementary Material.

Animal-derived models often incorrectly represent human cardiac features and diverge in stress-responses (Olson et al., 2000; Davis et al., 2011). hPSC-CMs offer an invaluable tool to study the human heart *in vitro*, provided that stress-responsive phenotypes are apparent and representative of *in vivo* conditions. The necessity of hPSC-CM priming for pathology modeling is apparent in some hereditary monogenic pathologies (Kim et al., 2013), but becomes essential for most non-genetic diseases described here, given their incidence later in adult life. Optimization of cardiac maturation and metabolic priming protocols generated better insight into the crosstalk between structural, functional and metabolic states of hPSC-CMs. These advances now allow more representative modeling of non-genetic diseases, still lagging behind the highly penetrant genetic conditions with clear analytical read-outs that dominate hPSC-CM literature (Figure 2A).

Currently, advanced modeling of the adult myocardium requires hEHTs. These multiparametric setups integrating stimulation and data acquisition systems, act as human preclinical models refining the predictive efficacy of less throughput-limited 2D hPSC-CM models (Fiedler et al., 2019). Nevertheless, while closely resembling adult tissue transcriptomic and functional features, hEHTs fall short of gaining the status of full-fledged *organoids*, not fully mimicking adult myocardial macroscopic ultrastructure (Tiburcy et al., 2017; Ronaldson-Bouchard et al., 2018), thus requiring additional bioengineering efforts to scale up the systems from tissue- to organ-models, as the recently proposed atrioventricular composite (Zhao et al., 2019).

Importantly, the widespread use of commercially available cell products in the studies reported here (Supplementary Table S1) highlights the necessity of increasing robustness and reproducibility of the results through differentiation and culture protocols standardization. Indeed, whenever patient-specificity is not essential, employment of standardized experimental platforms is desirable to study a plethora of environmental cardiac insults (Turnbull et al., 2018; Figure 2B), remaining mindful of the pitfalls of broadening the results of few cell lines to the general population and of the aspirations toward personalized medicine approaches.

Finally, combining hPSC-CM-based models with high precision genome-editing technologies will be instrumental in not only supporting modeling of hereditary diseases by screening artificially introduced genetic variants of unknown significance (VUSs) (Figure 2A), but also in dissecting complex dynamics between non-genetic pathological stimuli and genetic backgrounds characterized by polygenic interactions.

## Author Contributions

All authors reviewed the literature, wrote, edited, and approved the final version of the manuscript.

## Conflict of Interest

The authors declare that the research was conducted in the absence of any commercial or financial relationships that could be construed as a potential conflict of interest.
