# Supplementary material for: Beyond Family: Modeling Non-hereditary Heart Diseases With Human Pluripotent Stem Cell-Derived Cardiomyocytes
Source: Front Physiol. 2020 Apr 22;11:384. doi: 10.3389/fphys.2020.00384 (PMC7188911; doi:10.3389/fphys.2020.00384)
Supplement: Supplementary file 1 [file Data_Sheet_1.PDF]

Beyond Family: modeling non-hereditary heart diseases with human pluripotent stem cell-derived cardiomyocytes

Authors: *Sebastian Martewicz*[1*], *Michael Magnussen*[2], *Nicola Elvassore*[1,2,3,4,*]

[1] Shanghai Institute for Advanced Immunochemical Studies (SIAIS), ShanghaiTech University, 393 Middle Huaxia Road, Pudong New District, Shanghai 201210, China

[2] Stem Cells & Regenerative Medicine Section, UCL Great Ormond Street Institute of Child Health, 30 Guilford Street, London WC1N 1EH, UK

[3] Venetian Institute of Molecular Medicine, via Orus 2, 35129 Padova, Italy

[4] Department of Industrial Engineering, University of Padova, via Marzolo 9, 35131 Padova, Italy

*****Corresponding authors address:

E-mail: smartewicz@shanghaitech.edu.cn
Shanghai Institute for Advanced Immunochemical Studies
ShanghaiTech University
Room A401, Y-Building
393 Middle Huaxia Road, Shanghai 201210, China

E-mail: nicola.elvassore@unipd.it
Shanghai Institute for Advanced Immunochemical Studies
ShanghaiTech University
Room A407, Y-Building
393 Middle Huaxia Road, Shanghai 201210, China
Telephone: +39 049 827 5469
Fax: +39 049 827 7599

SUPPLEMENTARY MATERIALS

Supplementary Table 1 Detailed description of all non-genetic disease models reported in the review

Keller protocol: according to Yang t al., 2008 and Kattman et al., 2011

Mummery protocol: according to Mummery et al., 2003

Murry protocol: according to Laflamme et al., 2007

Palecek protocol: according to Lian et al., 2012

Wu protocol: according to Burridge et al., 2014

Own protocol: see reference for details

Metabolic selection: according to Tohyama et al., 2013

Metabolic maturation: see reference for details

Highlighted: studies employing commercially available hPSC-CMs or differentiation media

Condition	Reference	Differentiation	Treatment	Subject of the study
Ischemia	Hsieh et al., 2015	Keller protocol	Hypoxic chamber: 2h 0.1% pO_2 (no glucose, 20 mM lactate) + 2h 20% pO_2	$NaNO_2$/hypoxia-pretreatment cardioprotection
	Wei et al., 2017	Palecek protocol	Hypoxic chamber: 45min 0% pO_2 (no glucose) + 3h 95% pO_2	Dansheng injection cardioprotection
	Hidalgo et al., 2018	Palecek protocol + Metabolic maturation	Hypoxic chamber: 2h 0% pO_2 (no glucose, low pH) + 4h 20% pO_2	Cardioprotective drug testing in metabolically matured cells
	Kirby et al., 2018	Commercial hPSC-CMs	30min 300 µM or 60 µM H_2O_2 (no glucose)	High-throughput cardioprotective drug screening
	Martewicz et al., 2018	Palecek protocol	Microfluidic chip: 3h 0% pO_2 (no glucose) + 3h 20% pO_2	IR-resistance of unprimed and glycogen depleted CMs
	Zhao et al., 2019	Palecek protocol	Anaerobic pouch: 24h	Transcriptomic hypoxia-responses in human and macaque monkey CMs
	Chen and Vunjak-Novakovic, 2019	Wu protocol + EHT	Hypoxic chamber: 6h 0% pO_2 (no glucose, 20 mM lactate) + 3h 20% pO_2	Cardioprotective drug testing and ischemic pre-conditioning
	Fernández-Morales et al., 2019	Palecek protocol	Perfusion pipette proximity puff (low pH)	O_2- and low pH-mediated regulation of CaV1.2 function
	Fiedler et al., 2019	Commercial hPSC-CMs	H_2O_2 and menadione at varying concentrations and timings; hypoxia 3 cycles of 0.1% pO_2, over a period of 8 h	High-throughput cardioprotective drug screening and lead compound optimization
	Lu et al., 2019	Commercial hPSC-CMs	Hypoxic chamber: 24h 0% pO_2 + 6/12/24h 95% pO_2	Sevoflurane cardioprotection through apoptosis and autophagy modulation
	Mo et al., 2019	Wu protocol	Hypoxic chamber: 24h 1% pO_2 (no glucose)	Role of miR-30e-5p in cardiac hypoxia-response
	Sebastiao et al., 2019	Own protocol + Maturation medium	Hypoxic chamber: 5h 0% pO_2 (no glucose, 20 mM lactate) + 16h 3% pO_2	Paracrine crosstalk between CMs and cardiac progenitor cells
	Ward and Gilad,	Wu protocol + Metabolic	Hypoxic chamber: 6h 1% pO_2 (no glucose) + 6/24h 10%	Transcriptomic hypoxia-responses in human and chimpanzee CMs

	2019	selection	pO_2	
	Sebastiao et al., 2020	Own protocol + Maturation medium	Hypoxic chamber: 5h 0% pO_2 (no glucose, 20 mM lactate) + 16h 3% pO_2	Paracrine crosstalk between CMs and cardiac progenitor cells
Hypertrophy	Gao et al. 2011	Not specified	24h AngII 200 nM	SCN5A loss-of-function transcript expression in failing hearts
	Foldes et al., 2011	Spontaneous EB differentiation; Murry protocol	48h 10 µM PE; 48h 1 µM AngII; 24h equiaxial 10–25% stretch at 0.5 Hz	Description of hypertrophic phenotype
	Gao et al. 2013	Modified Murry protocol	48h AngII 200 nM	Unfolded Protein Response activation by SCN5A loss-of-function transcript expression in failing hearts
	Carlson et al., 2013	Commercial hPSC-CMs	18h ET-1 10 nM	HTS-compatible assay for anti-hypertrophic drugs
	Aggarwal et al., 2014	Commercial hPSC-CMs	18h ET-1 10 nM	Transcriptomic profiling of hypertrophic CMs
	Foldes et al., 2014	Spontaneous EB differentiation; Murry protocol	48h 10 µM PE; 24h 100 nM AngII; 24h ET-1 10 nM; 48h ISO 10 µM	Hypertrophic phenotype variability in hESC-/hiPSC-CMs and adrenergic response
	Martin et al., 2014	Commercial hPSC-CMs	24h ISO 10 µM	HSP20–PDE4D interaction in hypertrophic phenotype generation
	Tanaka et al., 2014	Spontaneous EB differentiation	7 days ET-1 various concentrations; 7 days AngII 100 nM; 7 days insulin-like growth factor 1 100 nM; 7 days PE 50 µM	Hypertrophic phenotype differences between healthy- and patient-derived hiPSC-CMs
	Cui et al., 2016	Palecek protocol + Metabolic selection	24h PE 10 µM; 24h ET-1 1 nM	Mechanism of hypertrophic signal transduction
	Mathieu et al., 2016	Commercial hPSC-CMs	24h AngII 100 nM	Sodium current (SCN5A) reduction in heart failure
	Wang et al., 2016	Mummery protocol	untreated	LncRNA in hypertrophic transcriptional program control
	Gesmundo et al., 2017	Spontaneous EB differentiation	24h PE 10 µM	Anti-hypertrophic effect of GHRH
	Nagai et al., 2017	Commercial hPSC-CMs	18h ET-1 10 nM	Anti-hypertrophic effect of small molecules
	Qin et al., 2017	Commercial hPSC-CMs	24h ET-1 1 nM	Anti-hypertrophic effect of small molecules
	Rupert et al., 2017	Palecek protocol	72h PE 2 µM + Timolol 200 nM	Description of hypertrophic phenotype analysis
	Tiburcy et al., 2017	Own protocol + Metabolic selection + EHT	7 days ET-1 10 nM; 7 days norepinephrine (various concentration)	Adrenergic response in hypertrophic model
	Zhang et al., 2017	Commercial hPSC-CMs	24h ET-1 10 nM	Anti-hypertrophic effect of danhong injection
	Ronaldson-Bouchard et al., 2018	Own protocol + EHT	24h AngII; 24h ET-1; 24h ISO	Description of hypertrophic phenotype in hEHTs
	Rosales et al., 2018	Commercial hPSC-CMs	24h AngII 200 µM; 24h ET-1 100 µM	Role of JMJD2A in hypertrophic phenotype generation
	Hoes et al., 2019	Wu protocol + Metabolic	24/48h equiaxial 15% stretch, 1 Hz	Cardioprotective role of cathepsin D

		selection		
	Mirtschink et al., 2019	Commercial hPSC-CMs	untreated	LncRNA in hypertrophic transcriptional program control
	Scrimgeour et al., 2019	Commercial hPSC-CMs	18h ET-1 10 nM	Role of miRNA-451a in hypertrophic phenotype generation
	Sewanan et al., 2019	Palecek protocol	untreated	hiPSC-CMs response to hypertrophic heart ECM
Diabetes	Sepac et al.,2010	Modified Murry protocol	50min $H_2O_2$10 mM	Isoflurane cardioprotection mechanism under oxidative stress
	Canfield et al., 2012	Modified Murry protocol	5/11/25 mM glucose	Isoflurane cardioprotection under hyperglycemic conditions
	Canfield et al., 2016	Murry protocol	5/11/25 mM glucose; 100 µM H_2O_2	Isoflurane cardioprotection mechanism under hyperglycemic conditions
	Chanda et al., 2017	Commercial differentiation kit	16h TNFα 10 ng/ml; 16h Palmitic acid 250 µM	Role of endocannabinoids in insulin resistance
	Drawnel et al., 2014	Commercial hPSC-CMs + Metabolic maturation	2 days glucose 10 mM + ET-1 10 nM + cortisol 1 µM	Description of diabetes-mimetic phenotype and drug screening
	Geraets et al.,2018	Commercial differentiation kit	16h 250 µM Palmitate + 50/100 nM insulin	Description of insulin-resistant phenotype
	Graneli et al.,2019	Commercial hPSC-CMs + Metabolic maturation	7 days glucose 20 mM + palmitate 50 µM + uric acid 150 µg/ml + linoleic acid 2x + ET-1 10 nM	Description of diabetes-mimetic phenotype
	Kikuchi et al., 2015	Palecek protocol	1h glucose 25 mM + insulin 100nM	Bioenergetics characterization and comparison with T1D-derived CMs
	Liu et al., 2017	Commercial differentiation kit	25h palmitate 20 µM + insulin 100 nM; 25h oleate 20 µM + insulin 100 nM	Mechanism of insulin resistance
	Ng et al., 2018	Spontaneous EB differentiation	14 days glucose 22 mM	In vitro follow-up on observed cardioprotection during clinical trial
	Wu et al.,2018	Not specified	2h 25 mM glucose + ET-1 50 nM	Cardioprotection by Oleanolic Acid treatment
	Pant et al., 2019	Commercial hPSC-CMs + Metabolic maturation	48h glucose 11 mM + ET-1 10 nM + cortisol 1 µM	Transcriptomic profiling of diabetes-mimetic CMs
Infectious Disease Model	Bozzi et al.,2019	Palecek protocol	T. cruzi infection	Description of cardiac phenotype in Chagas disease
	da Silva Lara et al., 2018	Commercial hPSC-CMs	T. cruzi infection	Description of cardiac phenotype in Chagas disease and drug treatment
	Sass et al., 2019a	Wu protocol	T. cruzi infection	Drug screening for Chagas disease
	Sass et al., 2019b	Wu protocol	T. cruzi infection	Drug screening for Chagas disease
	Scassa et al., 2011	Spontaneous EB differentiation	Coxsackievirus B infection	Description of viral infection in CMs
	Sharma et al.,2014	Palecek protocol + Modified metabolic selection	Coxsackievirus B3 infection	Description of viral infection in CMs and drug screening
	Yucel et al., 2017	Own protocol + Metabolic selection	48h lipopolysaccharide 100 µg/ml	Description of septic-like phenotype

Spaceflight Models	Acharya et al., 2019	Commercial hPSC-CMs	Parabolic flight, 30 cycles	Description of hypo- and hyper-gravity phenotype
	Becker et al., 2018a	Commercial hPSC-CMs	5 Gy X-ray irradiation	Transcriptomic characterization of irradiated CMs
	Becker et al., 2018b	Commercial hPSC-CMs	0-10 Gy X-ray irradiation	Electrophysiological characterization of irradiated CMs
	Wnorowski et al., 2019	Wu protocol	5.5 week culture on ISS	Description of CM phenotype after orbital flight
Fibrosis Models	Kumar et al., 2019	Palecek protocol + Metabolic selection	Hydrogel culture (various elastic moduli)	LncRNA in fibrotic phenotype evolution
	Zhang et al., 2019	Palecek protocol + Metabolic selection	Quiescence fibroblast co-culture	Crosstalk between CMs and fibroblasts
Other Pathology Models	Acun et al.,2019	Palecek protocol	Culture time extension; 16h H_2O_2 200 µM; Hypoxic chamber: 48h 1% pO2 + 24h 20% pO_2; Encapsulation in hydrogel (various elastic moduli)	Characterization of aging in vitro
	Gaber et al.,2013	Keller protocol	Hypoxic chamber: 72h 1% pO_2	Hypoplastic left heart syndrome modeling
	Lemme et al., 2019	Own protocol + EHT	Optical contraction pacing	Tachycardia modeling
	Naftali-Shani et al.,2018	Not specified	untreated	Description of peripartum cardiomyopathy phenotype
	Rampoldi et al.,2019	Own protocol	5 days ethanol 17/50/100/200 mM	Fetal ethanol exposure modeling
	Turnbull et al., 2019	Palecek protocol + EHT	Contact cryoinjury	Various cardiac damages
	Vogues et al., 2017	Own protocol	Contact cryoinjury	Description of early cardiac regenerative potential

Literature Research Methods.

PubMed database (https://www.ncbi.nlm.nih.gov/pubmed) was searched with the following queries. All search results were filtered out for “Review” category. The remaining hits were searched manually.

Ischemia

pluripotent[All Fields] AND ("myocytes, cardiac"[MeSH Terms] OR ("myocytes"[All Fields] AND "cardiac"[All Fields]) OR "cardiac myocytes"[All Fields] OR "cardiomyocyte"[All Fields] OR "cardiomyocytes"[All Fields]) AND ("ischaemia"[All Fields] OR "ischemia"[MeSH Terms] OR "ischemia"[All Fields] OR "hypoxia"[MeSH Terms] OR "hypoxia"[All Fields] OR "anoxia"[All Fields] OR "oxygen"[MeSH Terms] OR "oxygen"[All Fields] OR "infarction"[MeSH Terms] OR "infarction"[All Fields])

02Jan2020 462 hits (122 review)

Diabetes

pluripotent[All Fields] AND ("myocytes, cardiac"[MeSH Terms] OR ("myocytes"[All Fields] AND "cardiac"[All Fields]) OR "cardiac myocytes"[All Fields] OR "cardiomyocytes"[All Fields] OR "cardiomyocyte"[All Fields]) AND ("diabetes mellitus"[MeSH Terms] OR ("diabetes"[All Fields] AND "mellitus"[All Fields]) OR "diabetes mellitus"[All Fields] OR "diabetes"[All Fields] OR "diabetes insipidus"[MeSH Terms] OR ("diabetes"[All Fields] AND "insipidus"[All Fields]) OR "diabetes insipidus"[All Fields])

02Jan2020 53 hits (19 review)

Hypertrophy

(pluripotent[All Fields] AND ("myocytes, cardiac"[MeSH Terms] OR ("myocytes"[All Fields] AND "cardiac"[All Fields]) OR "cardiac myocytes"[All Fields] OR "cardiomyocyte"[All Fields] OR "cardiomyocytes"[All Fields])) AND ("hypertrophy"[MeSH Terms] OR "hypertrophy"[All Fields] OR "maladaptive"[All Fields])

02Jan2020 98 hits (8 review)

Fibrosis

(pluripotent[All Fields] AND ("myocytes, cardiac"[MeSH Terms] OR ("myocytes"[All Fields] AND "cardiac"[All Fields]) OR "cardiac myocytes"[All Fields] OR "cardiomyocyte"[All Fields] OR "cardiomyocytes"[All Fields])) AND ("fibrosis"[MeSH Terms] OR "fibrosis"[All Fields])

02Jan2020 67 hits (11 review)

Figure 2A search parameters:

(pluripotent[All Fields] AND ("myocytes, cardiac"[MeSH Terms] OR ("myocytes"[All Fields] AND "cardiac"[All Fields]) OR "cardiac myocytes"[All Fields] OR "cardiomyocyte"[All Fields] OR "cardiomyocytes"[All Fields])) AND ("2018"[PDAT] : "3000"[PDAT])

02Jan2020 843 hits (113 review)

References for Figure 2A

(Highlighted in red the studies employing Lab Engineered cell lines)

Familial Hypertrophic Cardiomyopathy (HCM)

1. Ben Jehuda, R., Eisen, B., Shemer, Y., Mekies, L.N., Szantai, A., Reiter, I., et al. (2018). CRISPR correction of the PRKAG2 gene mutation in the patient's induced pluripotent stem cell-derived cardiomyocytes eliminates electrophysiological and structural abnormalities. Heart Rhythm 15(2), 267-276. doi: 10.1016/j.hrthm.2017.09.024.
2. Chang, A.C.Y., Chang, A.C.H., Kirillova, A., Sasagawa, K., Su, W., Weber, G., et al. (2018). Telomere shortening is a hallmark of genetic cardiomyopathies. Proc Natl Acad Sci U S A 115(37), 9276-9281. doi: 10.1073/pnas.1714538115.
3. Cohn, R., Thakar, K., Lowe, A., Ladha, F.A., Pettinato, A.M., Romano, R., et al. (2019). A Contraction Stress Model of Hypertrophic Cardiomyopathy due to Sarcomere Mutations. Stem Cell Reports 12(1), 71-83. doi: 10.1016/j.stemcr.2018.11.015.
4. Dementyeva, E.V., Medvedev, S.P., Kovalenko, V.R., Vyatkin, Y.V., Kretov, E.I., Slotvitsky, M.M., et al. (2019). Applying Patient-Specific Induced Pluripotent Stem Cells to Create a Model of Hypertrophic Cardiomyopathy. Biochemistry (Mosc) 84(3), 291-298. doi: 10.1134/s0006297919030118.
5. Hallas, T., Eisen, B., Shemer, Y., Ben Jehuda, R., Mekies, L.N., Naor, S., et al. (2018). Investigating the cardiac pathology of SCO2-mediated hypertrophic cardiomyopathy using patients induced pluripotent stem cell-derived cardiomyocytes. J Cell Mol Med 22(2), 913-925. doi: 10.1111/jcmm.13392.
6. Helms, A.S., Tang, V.T., O'Leary, T.S., Friedline, S., Wauchope, M., Arora, A., et al. (2019). Effects of MYBPC3 loss of function mutations preceding hypertrophic cardiomyopathy. JCI Insight. doi: 10.1172/jci.insight.133782.
7. Hildebrandt, M.R., Reuter, M.S., Wei, W., Tayebi, N., Liu, J., Sharmin, S., et al. (2019). Precision Health Resource of Control iPSC Lines for Versatile Multilineage Differentiation. Stem Cell Reports 13(6), 1126-1141. doi: 10.1016/j.stemcr.2019.11.003.
8. Li, S., Pan, H., Tan, C., Sun, Y., Song, Y., Zhang, X., et al. (2018). Mitochondrial Dysfunctions Contribute to Hypertrophic Cardiomyopathy in Patient iPSC-Derived Cardiomyocytes with MT-RNR2 Mutation. Stem Cell Reports 10(3), 808-821. doi: 10.1016/j.stemcr.2018.01.013.
9. Li, X., Lu, W.J., Li, Y., Wu, F., Bai, R., Ma, S., et al. (2019). MLP-deficient human pluripotent stem cell derived cardiomyocytes develop hypertrophic cardiomyopathy and heart failure phenotypes due to abnormal calcium handling. Cell Death Dis 10(8), 610. doi: 10.1038/s41419-019-1826-4.
10. Lv, W., Qiao, L., Petrenko, N., Li, W., Owens, A.T., McDermott-Roe, C., et al. (2018). Functional Annotation of TNNT2 Variants of Uncertain Significance With Genome-Edited Cardiomyocytes. Circulation 138(24), 2852-2854. doi: 10.1161/circulationaha.118.035028.
11. Ma, N., Zhang, J.Z., Itzhaki, I., Zhang, S.L., Chen, H., Haddad, F., et al. (2018a). Determining the Pathogenicity of a Genomic Variant of Uncertain Significance Using CRISPR/Cas9 and Human-Induced Pluripotent Stem Cells. Circulation 138(23), 2666-2681. doi: 10.1161/circulationaha.117.032273.
12. Ma, Z., Huebsch, N., Koo, S., Mandegar, M.A., Siemons, B., Boggess, S., et al. (2018b).

Contractile deficits in engineered cardiac microtissues as a result of MYBPC3 deficiency and mechanical overload. Nat Biomed Eng 2(12), 955-967. doi: 10.1038/s41551-018-0280-4.
13. Mosqueira, D., Mannhardt, I., Bhagwan, J.R., Lis-Slimak, K., Katili, P., Scott, E., et al. (2018). CRISPR/Cas9 editing in human pluripotent stem cell-cardiomyocytes highlights arrhythmias, hypocontractility, and energy depletion as potential therapeutic targets for hypertrophic cardiomyopathy. Eur Heart J 39(43), 3879-3892. doi: 10.1093/eurheartj/ehy249.
14. Prajapati, C., Ojala, M., and Aalto-Setala, K. (2018). Divergent effects of adrenaline in human induced pluripotent stem cell-derived cardiomyocytes obtained from hypertrophic cardiomyopathy. Dis Model Mech 11(2). doi: 10.1242/dmm.032896.
15. Prondzynski, M., Lemoine, M.D., Zech, A.T., Horvath, A., Di Mauro, V., Koivumaki, J.T., et al. (2019). Disease modeling of a mutation in alpha-actinin 2 guides clinical therapy in hypertrophic cardiomyopathy. EMBO Mol Med 11(12), e11115. doi: 10.15252/emmm.201911115.
16. Sakai, T., Naito, A.T., Kuramoto, Y., Ito, M., Okada, K., Higo, T., et al. (2018). Phenotypic Screening Using Patient-Derived Induced Pluripotent Stem Cells Identified Pyr3 as a Candidate Compound for the Treatment of Infantile Hypertrophic Cardiomyopathy. Int Heart J 59(5), 1096-1105. doi: 10.1536/ihj.17-730.
17. Seeger, T., Shrestha, R., Lam, C.K., Chen, C., McKeithan, W.L., Lau, E., et al. (2019). A Premature Termination Codon Mutation in MYBPC3 Causes Hypertrophic Cardiomyopathy via Chronic Activation of Nonsense-Mediated Decay. Circulation 139(6), 799-811. doi: 10.1161/circulationaha.118.034624.
18. Smith, J.G.W., Owen, T., Bhagwan, J.R., Mosqueira, D., Scott, E., Mannhardt, I., et al. (2018). Isogenic Pairs of hiPSC-CMs with Hypertrophic Cardiomyopathy/LVNC-Associated ACTC1 E99K Mutation Unveil Differential Functional Deficits. Stem Cell Reports 11(5), 1226-1243. doi: 10.1016/j.stemcr.2018.10.006.
19. Tang, L., Yao, F., Wang, H., Wang, X., Shen, J., Dai, B., et al. (2019). Inhibition of TRPC1 prevents cardiac hypertrophy via NF-kappaB signaling pathway in human pluripotent stem cell-derived cardiomyocytes. J Mol Cell Cardiol 126, 143-154. doi: 10.1016/j.yjmcc.2018.10.020.
20. Toepfer, C.N., Sharma, A., Cicconet, M., Garfinkel, A.C., Mucke, M., Neyazi, M., et al. (2019). SarcTrack. Circ Res 124(8), 1172-1183. doi: 10.1161/circresaha.118.314505.
21. Viswanathan, S.K., Puckelwartz, M.J., Mehta, A., Ramachandra, C.J.A., Jagadeesan, A., Fritsche-Danielson, R., et al. (2018). Association of Cardiomyopathy With MYBPC3 D389V and MYBPC3Delta25bpIntronic Deletion in South Asian Descendants. JAMA Cardiol 3(6), 481-488. doi: 10.1001/jamacardio.2018.0618.
22. Wang, L., Kim, K., Parikh, S., Cadar, A.G., Bersell, K.R., He, H., et al. (2018). Hypertrophic cardiomyopathy-linked mutation in troponin T causes myofibrillar disarray and pro-arrhythmic action potential changes in human iPSC cardiomyocytes. J Mol Cell Cardiol 114, 320-327. doi: 10.1016/j.yjmcc.2017.12.002.
23. Wu, H., Yang, H., Rhee, J.W., Zhang, J.Z., Lam, C.K., Sallam, K., et al. (2019). Modelling diastolic dysfunction in induced pluripotent stem cell-derived cardiomyocytes from hypertrophic cardiomyopathy patients. Eur Heart J 40(45), 3685-3695. doi: 10.1093/eurheartj/ehz326.
24. Yang, K.C., Breitbart, A., De Lange, W.J., Hofsteen, P., Futakuchi-Tsuchida, A., Xu, J., et al.

(2018). Novel Adult-Onset Systolic Cardiomyopathy Due to MYH7 E848G Mutation in Patient-Derived Induced Pluripotent Stem Cells. JACC Basic Transl Sci 3(6), 728-740. doi: 10.1016/j.jacbts.2018.08.008.
25. Zhan, Y., Sun, X., Li, B., Cai, H., Xu, C., Liang, Q., et al. (2018). Establishment of a PRKAG2 cardiac syndrome disease model and mechanism study using human induced pluripotent stem cells. J Mol Cell Cardiol 117, 49-61. doi: 10.1016/j.yjmcc.2018.02.007.
26. Zhou, W., Bos, J.M., Ye, D., Tester, D.J., Hrstka, S., Maleszewski, J.J., et al. (2019). Induced Pluripotent Stem Cell-Derived Cardiomyocytes from a Patient with MYL2-R58Q-Mediated Apical Hypertrophic Cardiomyopathy Show Hypertrophy, Myofibrillar Disarray, and Calcium Perturbations. J Cardiovasc Transl Res 12(5), 394-403. doi: 10.1007/s12265-019-09873-6.

Familial Dilated Cardiomyopathy (DCM)

1. Bertero, A., Fields, P.A., Smith, A.S.T., Leonard, A., Beussman, K., Sniadecki, N.J., et al. (2019). Chromatin compartment dynamics in a haploinsufficient model of cardiac laminopathy. J Cell Biol 218(9), 2919-2944. doi: 10.1083/jcb.201902117.
2. Ceholski, D.K., Turnbull, I.C., Kong, C.W., Koplev, S., Mayourian, J., Gorski, P.A., et al. (2018). Functional and transcriptomic insights into pathogenesis of R9C phospholamban mutation using human induced pluripotent stem cell-derived cardiomyocytes. J Mol Cell Cardiol 119, 147-154. doi: 10.1016/j.yjmcc.2018.05.007.
3. Deacon, D.C., Happe, C.L., Chen, C., Tedeschi, N., Manso, A.M., Li, T., et al. (2019). Combinatorial interactions of genetic variants in human cardiomyopathy. Nat Biomed Eng 3(2), 147-157. doi: 10.1038/s41551-019-0348-9.
4. Lee, J., Termglinchan, V., Diecke, S., Itzhaki, I., Lam, C.K., Garg, P., et al. (2019). Activation of PDGF pathway links LMNA mutation to dilated cardiomyopathy. Nature 572(7769), 335-340. doi: 10.1038/s41586-019-1406-x.
5. McDermott-Roe, C., Lv, W., Maximova, T., Wada, S., Bukowy, J., Marquez, M., et al. (2019). Investigation of a dilated cardiomyopathy-associated variant in BAG3 using genome-edited iPSC-derived cardiomyocytes. JCI Insight 4(22). doi: 10.1172/jci.insight.128799.
6. Moreau, A., Gosselin-Badaroudine, P., Mercier, A., Burger, B., Keller, D.I., and Chahine, M. (2018). A leaky voltage sensor domain of cardiac sodium channels causes arrhythmias associated with dilated cardiomyopathy. Sci Rep 8(1), 13804. doi: 10.1038/s41598-018-31772-0.
7. Salvarani, N., Crasto, S., Miragoli, M., Bertero, A., Paulis, M., Kunderfranco, P., et al. (2019). The K219T-Lamin mutation induces conduction defects through epigenetic inhibition of SCN5A in human cardiac laminopathy. Nat Commun 10(1), 2267. doi: 10.1038/s41467-019-09929-w.
8. Schick, R., Mekies, L.N., Shemer, Y., Eisen, B., Hallas, T., Ben Jehuda, R., et al. (2018). Functional abnormalities in induced Pluripotent Stem Cell-derived cardiomyocytes generated from titin-mutated patients with dilated cardiomyopathy. PLoS One 13(10), e0205719. doi: 10.1371/journal.pone.0205719.
9. Shah, D., Virtanen, L., Prajapati, C., Kiamehr, M., Gullmets, J., West, G., et al. (2019). Modeling of LMNA-Related Dilated Cardiomyopathy Using Human Induced Pluripotent Stem Cells. Cells 8(6). doi: 10.3390/cells8060594.
10. Stroik, D.R., Ceholski, D.K., Bidwell, P.A., Mleczko, J., Thanel, P.F., Kamdar, F., et al. (2019).

Viral expression of a SERCA2a-activating PLB mutant improves calcium cycling and synchronicity in dilated cardiomyopathic hiPSC-CMs. J Mol Cell Cardiol 138, 59-65. doi: 10.1016/j.yjmcc.2019.11.147.

11. Zaunbrecher, R.J., Abel, A.N., Beussman, K., Leonard, A., von Frieling-Salewsky, M., Fields, P.A., et al. (2019). Cronos Titin Is Expressed in Human Cardiomyocytes and Necessary for Normal Sarcomere Function. Circulation 140(20), 1647-1660. doi: 10.1161/circulationaha.119.039521.

Familial Arrhythmogenic Cardiomyopathy (AC)

1. Blazeski, A., Lowenthal, J., Wang, Y., Teuben, R., Zhu, R., Gerecht, S., et al. (2019). Engineered Heart Slice Model of Arrhythmogenic Cardiomyopathy Using Plakophilin-2 Mutant Myocytes. Tissue Eng Part A 25(9-10), 725-735. doi: 10.1089/ten.TEA.2018.0272.
2. Chelko, S.P., Asimaki, A., Lowenthal, J., Bueno-Beti, C., Bedja, D., Scalco, A., et al. (2019). Therapeutic Modulation of the Immune Response in Arrhythmogenic Cardiomyopathy. Circulation 140(18), 1491-1505. doi: 10.1161/circulationaha.119.040676.
3. El-Battrawy, I., Zhao, Z., Lan, H., Cyganek, L., Tombers, C., Li, X., et al. (2018). Electrical dysfunctions in human-induced pluripotent stem cell-derived cardiomyocytes from a patient with an arrhythmogenic right ventricular cardiomyopathy. Europace 20(Fi1), f46-f56. doi: 10.1093/europace/euy042.
4. Khudiakov, A.A., Smolina, N.A., Perepelina, K.I., Malashicheva, A.B., and Kostareva, A.A. (2019). Extracellular MicroRNAs and Mitochondrial DNA as Potential Biomarkers of Arrhythmogenic Cardiomyopathy. Biochemistry (Mosc) 84(3), 272-282. doi: 10.1134/s000629791903009x.
5. Martewicz, S., Luni, C., Serena, E., Pavan, P., Chen, H.V., Rampazzo, A., et al. (2019). Transcriptomic Characterization of a Human In Vitro Model of Arrhythmogenic Cardiomyopathy Under Topological and Mechanical Stimuli. Ann Biomed Eng 47(3), 852-865. doi: 10.1007/s10439-018-02134-8.
6. Padron-Barthe, L., Villalba-Orero, M., Gomez-Salinero, J.M., Dominguez, F., Roman, M., Larrasa-Alonso, J., et al. (2019). Severe Cardiac Dysfunction and Death Caused by Arrhythmogenic Right Ventricular Cardiomyopathy Type 5 Are Improved by Inhibition of Glycogen Synthase Kinase-3beta. Circulation 140(14), 1188-1204. doi: 10.1161/circulationaha.119.040366.

Familial Long QT Syndrome (LQTS)

1. Blinova, K., Schocken, D., Patel, D., Daluwatte, C., Vicente, J., Wu, J.C., et al. (2019). Clinical Trial in a Dish: Personalized Stem Cell-Derived Cardiomyocyte Assay Compared With Clinical Trial Results for Two QT-Prolonging Drugs. Clin Transl Sci 12(6), 687-697. doi: 10.1111/cts.12674.
2. Chai, S., Wan, X., Ramirez-Navarro, A., Tesar, P.J., Kaufman, E.S., Ficker, E., et al. (2018). Physiological genomics identifies genetic modifiers of long QT syndrome type 2 severity. J Clin Invest 128(3), 1043-1056. doi: 10.1172/jci94996.
3. Chavali, N.V., Kryshtal, D.O., Parikh, S.S., Wang, L., Glazer, A.M., Blackwell, D.J., et al. (2019). Patient-independent human induced pluripotent stem cell model: A new tool for rapid determination of genetic variant pathogenicity in long QT syndrome. Heart Rhythm 16(11),

1686-1695. doi: 10.1016/j.hrthm.2019.04.031.
4. Estes, S.I., Ye, D., Zhou, W., Dotzler, S.M., Tester, D.J., Bos, J.M., et al. (2019). Characterization of the CACNA1C-R518C Missense Mutation in the Pathobiology of Long-QT Syndrome Using Human Induced Pluripotent Stem Cell Cardiomyocytes Shows Action Potential Prolongation and L-Type Calcium Channel Perturbation. Circ Genom Precis Med 12(8), e002534. doi: 10.1161/circgen.119.002534.
5. Garg, P., Oikonomopoulos, A., Chen, H., Li, Y., Lam, C.K., Sallam, K., et al. (2018). Genome Editing of Induced Pluripotent Stem Cells to Decipher Cardiac Channelopathy Variant. J Am Coll Cardiol 72(1), 62-75. doi: 10.1016/j.jacc.2018.04.041.
6. Hall, A.R., Anderson, C.L., Smith, J.L., Mirshahi, T., Elayi, C.S., January, C.T., et al. (2018). Visualizing Mutation-Specific Differences in the Trafficking-Deficient Phenotype of Kv11.1 Proteins Linked to Long QT Syndrome Type 2. Front Physiol 9, 584. doi: 10.3389/fphys.2018.00584.
7. Kroncke, B.M., Yang, T., and Roden, D.M. (2019). Multiple mechanisms underlie increased cardiac late sodium current. Heart Rhythm 16(7), 1091-1097. doi: 10.1016/j.hrthm.2019.01.018.
8. Mehta, A., Ramachandra, C.J.A., Singh, P., Chitre, A., Lua, C.H., Mura, M., et al. (2018). Identification of a targeted and testable antiarrhythmic therapy for long-QT syndrome type 2 using a patient-specific cellular model. Eur Heart J 39(16), 1446-1455. doi: 10.1093/eurheartj/ehx394.
9. Mesquita, F.C.P., Arantes, P.C., Kasai-Brunswick, T.H., Araujo, D.S., Gubert, F., Monnerat, G., et al. (2019). R534C mutation in hERG causes a trafficking defect in iPSC-derived cardiomyocytes from patients with type 2 long QT syndrome. Sci Rep 9(1), 19203. doi: 10.1038/s41598-019-55837-w.
10. Mura, M., Ginevrino, M., Zappatore, R., Pisano, F., Boni, M., Castelletti, S., et al. (2018a). Generation of the human induced pluripotent stem cell (hiPSC) line PSMi003-A from a patient affected by an autosomal recessive form of Long QT Syndrome type 1. Stem Cell Res 29, 170-173. doi: 10.1016/j.scr.2018.04.003.
11. Mura, M., Lee, Y.K., Ginevrino, M., Zappatore, R., Pisano, F., Boni, M., et al. (2018b). Generation of the human induced pluripotent stem cell (hiPSC) line PSMi002-A from a patient affected by the Jervell and Lange-Nielsen syndrome and carrier of two compound heterozygous mutations on the KCNQ1 gene. Stem Cell Res 29, 157-161. doi: 10.1016/j.scr.2018.04.002.
12. Mura, M., Lee, Y.K., Pisano, F., Ginevrino, M., Boni, M., Calabro, F., et al. (2019a). Generation of the human induced pluripotent stem cell (hiPSC) line PSMi004-A from a carrier of the KCNQ1-R594Q mutation. Stem Cell Res 37, 101431. doi: 10.1016/j.scr.2019.101431.
13. Mura, M., Lee, Y.K., Pisano, F., Ginevrino, M., Boni, M., Calabro, F., et al. (2019b). Generation of the human induced pluripotent stem cell (hiPSC) line PSMi005-A from a patient carrying the KCNQ1-R190W mutation. Stem Cell Res 37, 101437. doi: 10.1016/j.scr.2019.101437.
14. Mura, M., Pisano, F., Stefanello, M., Ginevrino, M., Boni, M., Calabro, F., et al. (2019c). Generation of the human induced pluripotent stem cell (hiPSC) line PSMi007-A from a Long QT Syndrome type 1 patient carrier of two common variants in the NOS1AP gene. Stem Cell Res 36, 101416. doi: 10.1016/j.scr.2019.101416.
15. Pahlavan, S., Tousi, M.S., Ayyari, M., Alirezalu, A., Ansari, H., Saric, T., et al. (2018). Effects of

hawthorn (Crataegus pentagyna) leaf extract on electrophysiologic properties of cardiomyocytes derived from human cardiac arrhythmia-specific induced pluripotent stem cells. Faseb j 32(3), 1440-1451. doi: 10.1096/fj.201700494RR.
16. Perry, M.D., Ng, C.A., Mangala, M.M., Ng, T.Y.M., Hines, A.D., Liang, W., et al. (2019). Pharmacological activation of IKr in models of long QT Type 2 risks overcorrection of repolarization. Cardiovasc Res. doi: 10.1093/cvr/cvz247.
17. Schwartz, P.J., Gnecchi, M., Dagradi, F., Castelletti, S., Parati, G., Spazzolini, C., et al. (2019). From patient-specific induced pluripotent stem cells to clinical translation in long QT syndrome Type 2. Eur Heart J 40(23), 1832-1836. doi: 10.1093/eurheartj/ehz023.
18. Takaki, T., Inagaki, A., Chonabayashi, K., Inoue, K., Miki, K., Ohno, S., et al. (2019). Optical Recording of Action Potentials in Human Induced Pluripotent Stem Cell-Derived Cardiac Single Cells and Monolayers Generated from Long QT Syndrome Type 1 Patients. Stem Cells Int 2019, 7532657. doi: 10.1155/2019/7532657.
19. Wang, Z., Wang, L., Liu, W., Hu, D., Gao, Y., Ge, Q., et al. (2019). Pathogenic mechanism and gene correction for LQTS-causing double mutations in KCNQ1 using a pluripotent stem cell model. Stem Cell Res 38, 101483. doi: 10.1016/j.scr.2019.101483.
20. Wei, H., Wu, J., and Liu, Z. (2018). Studying KCNQ1 Mutation and Drug Response in Type 1 Long QT Syndrome Using Patient-Specific Induced Pluripotent Stem Cell-Derived Cardiomyocytes. Methods Mol Biol 1684, 7-28. doi: 10.1007/978-1-4939-7362-0_2.
21. Wuriyanghai, Y., Makiyama, T., Sasaki, K., Kamakura, T., Yamamoto, Y., Hayano, M., et al. (2018). Complex aberrant splicing in the induced pluripotent stem cell-derived cardiomyocytes from a patient with long QT syndrome carrying KCNQ1-A344Aspl mutation. Heart Rhythm 15(10), 1566-1574. doi: 10.1016/j.hrthm.2018.05.028.
22. Yoshinaga, D., Baba, S., Makiyama, T., Shibata, H., Hirata, T., Akagi, K., et al. (2019). Phenotype-Based High-Throughput Classification of Long QT Syndrome Subtypes Using Human Induced Pluripotent Stem Cells. Stem Cell Reports 13(2), 394-404. doi: 10.1016/j.stemcr.2019.06.007.

Familial Short QT Syndrome (SQTS)

1. El-Battrawy, I., Lan, H., Cyganek, L., Zhao, Z., Li, X., Buljubasic, F., et al. (2018). Modeling Short QT Syndrome Using Human-Induced Pluripotent Stem Cell-Derived Cardiomyocytes. J Am Heart Assoc 7(7). doi: 10.1161/jaha.117.007394.
2. Guo, F., Sun, Y., Wang, X., Wang, H., Wang, J., Gong, T., et al. (2019). Patient-Specific and Gene-Corrected Induced Pluripotent Stem Cell-Derived Cardiomyocytes Elucidate Single-Cell Phenotype of Short QT Syndrome. Circ Res 124(1), 66-78. doi: 10.1161/circresaha.118.313518.
3. Shinnawi, R., Shaheen, N., Huber, I., Shiti, A., Arbel, G., Gepstein, A., et al. (2019). Modeling Reentry in the Short QT Syndrome With Human-Induced Pluripotent Stem Cell-Derived Cardiac Cell Sheets. J Am Coll Cardiol 73(18), 2310-2324. doi: 10.1016/j.jacc.2019.02.055.
4. Zhao, Z., Li, X., El-Battrawy, I., Lan, H., Zhong, R., Xu, Q., et al. (2019). Drug Testing in Human-Induced Pluripotent Stem Cell-Derived Cardiomyocytes From a Patient With Short QT Syndrome Type 1. Clin Pharmacol Ther 106(3), 642-651. doi: 10.1002/cpt.1449.

Catecholaminergic Polymorphic Ventricular Tachycardia (CPVT)

1. Acimovic, I., Refaat, M.M., Moreau, A., Salykin, A., Reiken, S., Sleiman, Y., et al. (2018). Post-Translational Modifications and Diastolic Calcium Leak Associated to the Novel RyR2-D3638A Mutation Lead to CPVT in Patient-Specific hiPSC-Derived Cardiomyocytes. J Clin Med 7(11). doi: 10.3390/jcm7110423.
2. Bezzerides, V.J., Caballero, A., Wang, S., Ai, Y., Hylind, R.J., Lu, F., et al. (2019). Gene Therapy for Catecholaminergic Polymorphic Ventricular Tachycardia by Inhibition of Ca(2+)/Calmodulin-Dependent Kinase II. Circulation 140(5), 405-419. doi: 10.1161/circulationaha.118.038514.
3. Mohamed, B.A., Hartmann, N., Tirilomis, P., Sekeres, K., Li, W., Neef, S., et al. (2018). Sarcoplasmic reticulum calcium leak contributes to arrhythmia but not to heart failure progression. Sci Transl Med 10(458). doi: 10.1126/scitranslmed.aan0724.
4. Park, S.J., Zhang, D., Qi, Y., Li, Y., Lee, K.Y., Bezzerides, V.J., et al. (2019). Insights Into the Pathogenesis of Catecholaminergic Polymorphic Ventricular Tachycardia From Engineered Human Heart Tissue. Circulation 140(5), 390-404. doi: 10.1161/circulationaha.119.039711.
5. Polonen, R.P., Penttinen, K., Swan, H., and Aalto-Setala, K. (2018). Antiarrhythmic Effects of Carvedilol and Flecainide in Cardiomyocytes Derived from Catecholaminergic Polymorphic Ventricular Tachycardia Patients. Stem Cells Int 2018, 9109503. doi: 10.1155/2018/9109503.
6. Polonen, R.P., Swan, H., and Aalto-Setala, K. (2019). Mutation-specific differences in arrhythmias and drug responses in CPVT patients: simultaneous patch clamp and video imaging of iPSC derived cardiomyocytes. Mol Biol Rep. doi: 10.1007/s11033-019-05201-y.
7. Wei, H., Zhang, X.H., Clift, C., Yamaguchi, N., and Morad, M. (2018). CRISPR/Cas9 Gene editing of RyR2 in human stem cell-derived cardiomyocytes provides a novel approach in investigating dysfunctional Ca(2+) signaling. Cell Calcium 73, 104-111. doi: 10.1016/j.ceca.2018.04.009.

Brugada Syndrome (BS)

1. Angsutararux, P., Luanpitpong, S., Chingsuwanrote, P., Supraditaporn, K., Waeteekul, S., Terbto, P., et al. (2019). Generation of human induced pluripotent stem cell line carrying SCN5AC2204>T Brugada mutation (MUSli009-A-1) introduced by CRISPR/Cas9-mediated genome editing. Stem Cell Res 41, 101618. doi: 10.1016/j.scr.2019.101618.
2. Belbachir, N., Portero, V., Al Sayed, Z.R., Gourraud, J.B., Dilasser, F., Jesel, L., et al. (2019). RRAD mutation causes electrical and cytoskeletal defects in cardiomyocytes derived from a familial case of Brugada syndrome. Eur Heart J 40(37), 3081-3094. doi: 10.1093/eurheartj/ehz308.
3. de la Roche, J., Angsutararux, P., Kempf, H., Janan, M., Bolesani, E., Thiemann, S., et al. (2019). Comparing human iPSC-cardiomyocytes versus HEK293T cells unveils disease-causing effects of Brugada mutation A735V of NaV1.5 sodium channels. Sci Rep 9(1), 11173. doi: 10.1038/s41598-019-47632-4.
4. El-Battrawy, I., Albers, S., Cyganek, L., Zhao, Z., Lan, H., Li, X., et al. (2019a). A cellular model of Brugada syndrome with SCN10A variants using human-induced pluripotent stem cell-derived cardiomyocytes. Europace 21(9), 1410-1421. doi: 10.1093/europace/euz122.
5. El-Battrawy, I., Muller, J., Zhao, Z., Cyganek, L., Zhong, R., Zhang, F., et al. (2019b). Studying Brugada Syndrome With an SCN1B Variants in Human-Induced Pluripotent Stem Cell-Derived Cardiomyocytes. Front Cell Dev Biol 7, 261. doi: 10.3389/fcell.2019.00261.

6. Ma, D., Liu, Z., Loh, L.J., Zhao, Y., Li, G., Liew, R., et al. (2018). Identification of an INa-dependent and Ito-mediated proarrhythmic mechanism in cardiomyocytes derived from pluripotent stem cells of a Brugada syndrome patient. Sci Rep 8(1), 11246. doi: 10.1038/s41598-018-29574-5.
7. Perez-Hernandez, M., Matamoros, M., Alfayate, S., Nieto-Marin, P., Utrilla, R.G., Tinaquero, D., et al. (2018). Brugada syndrome trafficking-defective Nav1.5 channels can trap cardiac Kir2.1/2.2 channels. JCI Insight 3(18). doi: 10.1172/jci.insight.96291.
8. Selga, E., Sendfeld, F., Martinez-Moreno, R., Medine, C.N., Tura-Ceide, O., Wilmut, S.I., et al. (2018). Sodium channel current loss of function in induced pluripotent stem cell-derived cardiomyocytes from a Brugada syndrome patient. J Mol Cell Cardiol 114, 10-19. doi: 10.1016/j.yjmcc.2017.10.002.

Familial Atrial Fibrillation (AF)

1. Benzoni, P., Campostrini, G., Landi, S., Bertini, V., Marchina, E., Iascone, M., et al. (2019). Human iPSC modeling of a familial form of atrial fibrillation reveals a gain of function of If and ICaL in patient-derived cardiomyocytes. Cardiovasc Res. doi: 10.1093/cvr/cvz217.
2. Ghazizadeh, Z., Kiviniemi, T.O., Olafsson, S., Plotnick, D., Beerens, M., Zhang, K., et al. (2019). A Metastable Atrial State Underlies The Primary Genetic Substrate for MYL4 Mutation-Associated Atrial Fibrillation. Circulation. doi: 10.1161/circulationaha.119.044268.

Muscular Dystrophies (DYS)

1. Aminzadeh, M.A., Rogers, R.G., Fournier, M., Tobin, R.E., Guan, X., Childers, M.K., et al. (2018). Exosome-Mediated Benefits of Cell Therapy in Mouse and Human Models of Duchenne Muscular Dystrophy. Stem Cell Reports 10(3), 942-955. doi: 10.1016/j.stemcr.2018.01.023.
2. Dinarelli, S., Girasole, M., Spitalieri, P., Talarico, R.V., Murdocca, M., Botta, A., et al. (2018). AFM nano-mechanical study of the beating profile of hiPSC-derived cardiomyocytes beating bodies WT and DM1. J Mol Recognit 31(10), e2725. doi: 10.1002/jmr.2725.
3. Eisen, B., Ben Jehuda, R., Cuttitta, A.J., Mekies, L.N., Reiter, I., Ramchandren, S., et al. (2018). Generation of Duchenne muscular dystrophy patient-specific induced pluripotent stem cell line lacking exons 45-50 of the dystrophin gene (IITi001-A). Stem Cell Res 29, 111-114. doi: 10.1016/j.scr.2018.03.023.
4. Eisen, B., Ben Jehuda, R., Cuttitta, A.J., Mekies, L.N., Shemer, Y., Baskin, P., et al. (2019). Electrophysiological abnormalities in induced pluripotent stem cell-derived cardiomyocytes generated from Duchenne muscular dystrophy patients. J Cell Mol Med 23(3), 2125-2135. doi: 10.1111/jcmm.14124.
5. El-Battrawy, I., Zhao, Z., Lan, H., Li, X., Yucel, G., Lang, S., et al. (2018). Ion Channel Dysfunctions in Dilated Cardiomyopathy in Limb-Girdle Muscular Dystrophy. Circ Genom Precis Med 11(3), e001893. doi: 10.1161/circgen.117.001893.
6. Farini, A., Gowran, A., Bella, P., Sitzia, C., Scopece, A., Castiglioni, E., et al. (2019). Fibrosis Rescue Improves Cardiac Function in Dystrophin-Deficient Mice and Duchenne Patient-Specific Cardiomyocytes by Immunoproteasome Modulation. Am J Pathol 189(2), 339-353. doi: 10.1016/j.ajpath.2018.10.010.
7. Kim, E.Y., Barefield, D.Y., Vo, A.H., Gacita, A.M., Schuster, E.J., Wyatt, E.J., et al. (2019).

Distinct pathological signatures in human cellular models of myotonic dystrophy subtypes. JCI Insight 4(6). doi: 10.1172/jci.insight.122686.
8. Long, C., Li, H., Tiburcy, M., Rodriguez-Caycedo, C., Kyrychenko, V., Zhou, H., et al. (2018). Correction of diverse muscular dystrophy mutations in human engineered heart muscle by single-site genome editing. Sci Adv 4(1), eaap9004. doi: 10.1126/sciadv.aap9004.
9. Min, Y.L., Li, H., Rodriguez-Caycedo, C., Mireault, A.A., Huang, J., Shelton, J.M., et al. (2019). CRISPR-Cas9 corrects Duchenne muscular dystrophy exon 44 deletion mutations in mice and human cells. Sci Adv 5(3), eaav4324. doi: 10.1126/sciadv.aav4324.
10. Pioner, J.M., Guan, X., Klaiman, J.M., Racca, A.W., Pabon, L., Muskheli, V., et al. (2019). Absence of full-length dystrophin impairs normal maturation and contraction of cardiomyocytes derived from human induced pluripotent stem cells. Cardiovasc Res. doi: 10.1093/cvr/cvz109.
11. Sato, M., Shiba, N., Miyazaki, D., Shiba, Y., Echigoya, Y., Yokota, T., et al. (2019). Amelioration of intracellular Ca(2+) regulation by exon-45 skipping in Duchenne muscular dystrophy-induced pluripotent stem cell-derived cardiomyocytes. Biochem Biophys Res Commun 520(1), 179-185. doi: 10.1016/j.bbrc.2019.09.095.
12. Spitalieri, P., Talarico, R.V., Caioli, S., Murdocca, M., Serafino, A., Girasole, M., et al. (2018). Modelling the pathogenesis of Myotonic Dystrophy type 1 cardiac phenotype through human iPSC-derived cardiomyocytes. J Mol Cell Cardiol 118, 95-109. doi: 10.1016/j.yjmcc.2018.03.012.
13. Tsurumi, F., Baba, S., Yoshinaga, D., Umeda, K., Hirata, T., Takita, J., et al. (2019). The intracellular Ca2+ concentration is elevated in cardiomyocytes differentiated from hiPSCs derived from a Duchenne muscular dystrophy patient. PLoS One 14(3), e0213768. doi: 10.1371/journal.pone.0213768.
14. Wang, Y., Hao, L., Wang, H., Santostefano, K., Thapa, A., Cleary, J., et al. (2018). Therapeutic Genome Editing for Myotonic Dystrophy Type 1 Using CRISPR/Cas9. Mol Ther 26(11), 2617-2630. doi: 10.1016/j.ymthe.2018.09.003.

Friedreich's Ataxia (FRDA)

1. Bolotta, A., Abruzzo, P.M., Baldassarro, V.A., Ghezzo, A., Scotlandi, K., Marini, M., et al. (2019). New Insights into the Hepcidin-Ferroportin Axis and Iron Homeostasis in iPSC-Derived Cardiomyocytes from Friedreich's Ataxia Patient. Oxid Med Cell Longev 2019, 7623023. doi: 10.1155/2019/7623023.
2. Lai, J.I., Nachun, D., Petrosyan, L., Throesch, B., Campau, E., Gao, F., et al. (2019). Transcriptional profiling of isogenic Friedreich ataxia neurons and effect of an HDAC inhibitor on disease signatures. J Biol Chem 294(6), 1846-1859. doi: 10.1074/jbc.RA118.006515.
3. Li, J., Rozwadowska, N., Clark, A., Fil, D., Napierala, J.S., and Napierala, M. (2019). Excision of the expanded GAA repeats corrects cardiomyopathy phenotypes of iPSC-derived Friedreich's ataxia cardiomyocytes. Stem Cell Res 40, 101529. doi: 10.1016/j.scr.2019.101529.
4. Wong, A.O., Wong, G., Shen, M., Chow, M.Z., Tse, W.W., Gurung, B., et al. (2019). Correlation between frataxin expression and contractility revealed by in vitro Friedreich's ataxia cardiac tissue models engineered from human pluripotent stem cells. Stem Cell Res Ther 10(1), 203. doi: 10.1186/s13287-019-1305-y.

Fabry Disease (FD)

1. Birket, M.J., Raibaud, S., Lettieri, M., Adamson, A.D., Letang, V., Cervello, P., et al. (2019). A Human Stem Cell Model of Fabry Disease Implicates LIMP-2 Accumulation in Cardiomyocyte Pathology. Stem Cell Reports 13(2), 380-393. doi: 10.1016/j.stemcr.2019.07.004.
2. Chien, Y., Chou, S.J., Chang, Y.L., Leu, H.B., Yang, Y.P., Tsai, P.H., et al. (2018). Inhibition of Arachidonate 12/15-Lipoxygenase Improves alpha-Galactosidase Efficacy in iPSC-Derived Cardiomyocytes from Fabry Patients. Int J Mol Sci 19(5). doi: 10.3390/ijms19051480.
3. Kuramoto, Y., Naito, A.T., Tojo, H., Sakai, T., Ito, M., Shibamoto, M., et al. (2018). Generation of Fabry cardiomyopathy model for drug screening using induced pluripotent stem cell-derived cardiomyocytes from a female Fabry patient. J Mol Cell Cardiol 121, 256-265. doi: 10.1016/j.yjmcc.2018.07.246.

Danon Disease (DD)

1. Chi, C., Leonard, A., Knight, W.E., Beussman, K.M., Zhao, Y., Cao, Y., et al. (2019). LAMP-2B regulates human cardiomyocyte function by mediating autophagosome-lysosome fusion. Proc Natl Acad Sci U S A 116(2), 556-565. doi: 10.1073/pnas.1808618116.
2. Yoshida, S., Nakanishi, C., Okada, H., Mori, M., Yokawa, J., Yoshimuta, T., et al. (2018). Characteristics of induced pluripotent stem cells from clinically divergent female monozygotic twins with Danon disease. J Mol Cell Cardiol 114, 234-242. doi: 10.1016/j.yjmcc.2017.11.019.

Noonan Syndrome (NS)

1. Higgins, E.M., Bos, J.M., Dotzler, S.M., John Kim, C.S., and Ackerman, M.J. (2019). MRAS Variants Cause Cardiomyocyte Hypertrophy in Patient-Specific Induced Pluripotent Stem Cell-Derived Cardiomyocytes: Additional Evidence for MRAS as a Definitive Noonan Syndrome-Susceptibility Gene. Circ Genom Precis Med 12(11), e002648. doi: 10.1161/circgen.119.002648.
2. Jaffre, F., Miller, C.L., Schanzer, A., Evans, T., Roberts, A.E., Hahn, A., et al. (2019). Inducible Pluripotent Stem Cell-Derived Cardiomyocytes Reveal Aberrant Extracellular Regulated Kinase 5 and Mitogen-Activated Protein Kinase Kinase 1/2 Signaling Concomitantly Promote Hypertrophic Cardiomyopathy in RAF1-Associated Noonan Syndrome. Circulation 140(3), 207-224. doi: 10.1161/circulationaha.118.037227.

Other Genetic Conditions (Other)

1. Brodehl, A., Pour Hakimi, S.A., Stanasiuk, C., Ratnavadivel, S., Hendig, D., Gaertner, A., et al. (2019). Restrictive Cardiomyopathy is Caused by a Novel Homozygous Desmin (DES) Mutation p.Y122H Leading to a Severe Filament Assembly Defect. Genes (Basel) 10(11). doi: 10.3390/genes10110918.
2. Cao, Y., Xu, J., Wen, J., Ma, X., Liu, F., Li, Y., et al. (2018). Generation of a Urine-Derived Ips Cell Line from a Patient with a Ventricular Septal Defect and Heart Failure and the Robust Differentiation of These Cells to Cardiomyocytes via Small Molecules. Cell Physiol Biochem 50(2), 538-551. doi: 10.1159/000494167.
3. Cartwright, J.H., Aziz, Q., Harmer, S.C., Thayyil, S., Tinker, A., and Munroe, P.B. (2019). Genetic variants in TRPM7 associated with unexplained stillbirth modify ion channel

function. Hum Mol Genet. doi: 10.1093/hmg/ddz198.
4. Chowdhury, A., Aich, A., Jain, G., Wozny, K., Luchtenborg, C., Hartmann, M., et al. (2018). Defective Mitochondrial Cardiolipin Remodeling Dampens HIF-1alpha Expression in Hypoxia. Cell Rep 25(3), 561-570.e566. doi: 10.1016/j.celrep.2018.09.057.
5. Fatica, E.M., DeLeonibus, G.A., House, A., Kodger, J.V., Pearce, R.W., Shah, R.R., et al. (2019). Barth Syndrome: Exploring Cardiac Metabolism with Induced Pluripotent Stem Cell-Derived Cardiomyocytes. Metabolites 9(12). doi: 10.3390/metabo9120306.
6. Frasier, C.R., Zhang, H., Offord, J., Dang, L.T., Auerbach, D.S., Shi, H., et al. (2018). Channelopathy as a SUDEP Biomarker in Dravet Syndrome Patient-Derived Cardiac Myocytes. Stem Cell Reports 11(3), 626-634. doi: 10.1016/j.stemcr.2018.07.012.
7. Galera-Monge, T., Zurita-Diaz, F., Garesse, R., and Gallardo, M.E. (2019). The mutation m.13513G>A impairs cardiac function, favoring a neuroectoderm commitment, in a mutant-load dependent way. J Cell Physiol 234(11), 19511-19522. doi: 10.1002/jcp.28549.
8. Lee, J.J., Cheng, S.J., Huang, C.Y., Chen, C.Y., Feng, L., Hwang, D.Y., et al. (2019). Primary cardiac manifestation of autosomal dominant polycystic kidney disease revealed by patient induced pluripotent stem cell-derived cardiomyocytes. EBioMedicine 40, 675-684. doi: 10.1016/j.ebiom.2019.01.011.
9. Parra, V., Altamirano, F., Hernandez-Fuentes, C.P., Tong, D., Kyrychenko, V., Rotter, D., et al. (2018). Down Syndrome Critical Region 1 Gene, Rcan1, Helps Maintain a More Fused Mitochondrial Network. Circ Res 122(6), e20-e33. doi: 10.1161/circresaha.117.311522.
10. Shafaattalab, S., Li, A.Y., Lin, E., Stevens, C.M., Dewar, L.J., Lynn, F.C., et al. (2019). In vitro analyses of suspected arrhythmogenic thin filament variants as a cause of sudden cardiac death in infants. Proc Natl Acad Sci U S A 116(14), 6969-6974. doi: 10.1073/pnas.1819023116.
11. Takasaki, A., Hirono, K., Hata, Y., Wang, C., Takeda, M., Yamashita, J.K., et al. (2018). Sarcomere gene variants act as a genetic trigger underlying the development of left ventricular noncompaction. Pediatr Res 84(5), 733-742. doi: 10.1038/s41390-018-0162-1.
12. Veerman, C.C., Mengarelli, I., Koopman, C.D., Wilders, R., van Amersfoorth, S.C., Bakker, D., et al. (2019). Genetic variation in GNB5 causes bradycardia by augmenting the cholinergic response via increased acetylcholine-activated potassium current (I K,ACh). Dis Model Mech 12(7). doi: 10.1242/dmm.037994.
13. Wren, L.M., Jimenez-Jaimez, J., Al-Ghamdi, S., Al-Aama, J.Y., Bdeir, A., Al-Hassnan, Z.N., et al. (2019). Genetic Mosaicism in Calmodulinopathy. Circ Genom Precis Med 12(9), 375-385. doi: 10.1161/circgen.119.002581.

Non-genetic Conditions (Non-genetic)

1. Acharya, A., Brungs, S., Lichterfeld, Y., Hescheler, J., Hemmersbach, R., Boeuf, H., et al. (2019). Parabolic, Flight-Induced, Acute Hypergravity and Microgravity Effects on the Beating Rate of Human Cardiomyocytes. Cells 8(4). doi: 10.3390/cells8040352.
2. Acun, A., Nguyen, T.D., and Zorlutuna, P. (2019). In vitro aged, hiPSC-origin engineered heart tissue models with age-dependent functional deterioration to study myocardial infarction. Acta Biomater 94, 372-391. doi: 10.1016/j.actbio.2019.05.064.
3. Becker, B.V., Majewski, M., Abend, M., Palnek, A., Nestler, K., Port, M., et al. (2018a). Gene expression changes in human iPSC-derived cardiomyocytes after X-ray irradiation. Int J

Radiat Biol 94(12), 1095-1103. doi: 10.1080/09553002.2018.1516908.
4. Becker, B.V., Seeger, T., Beiert, T., Antwerpen, M., Palnek, A., Port, M., et al. (2018b). Impact of Ionizing Radiation on Electrophysiological Behavior of Human-induced Ipsc-derived Cardiomyocytes on Multielectrode Arrays. Health Phys 115(1), 21-28. doi: 10.1097/hp.0000000000000817.
5. Bozzi, A., Sayed, N., Matsa, E., Sass, G., Neofytou, E., Clemons, K.V., et al. (2019). Using Human Induced Pluripotent Stem Cell-Derived Cardiomyocytes as a Model to Study Trypanosoma cruzi Infection. Stem Cell Reports 12(6), 1232-1241. doi: 10.1016/j.stemcr.2019.04.017.
6. Chen, T., and Vunjak-Novakovic, G. (2019). Human Tissue-Engineered Model of Myocardial Ischemia-Reperfusion Injury. Tissue Eng Part A 25(9-10), 711-724. doi: 10.1089/ten.TEA.2018.0212.
7. da Silva Lara, L., Andrade-Lima, L., Magalhaes Calvet, C., Borsoi, J., Lopes Alberto Duque, T., Henriques-Pons, A., et al. (2018). Trypanosoma cruzi infection of human induced pluripotent stem cell-derived cardiomyocytes: an in vitro model for drug screening for Chagas disease. Microbes Infect 20(5), 312-316. doi: 10.1016/j.micinf.2018.03.002.
8. Fernandez-Morales, J.C., Hua, W., Yao, Y., and Morad, M. (2019). Regulation of Ca(2+) signaling by acute hypoxia and acidosis in cardiomyocytes derived from human induced pluripotent stem cells. Cell Calcium 78, 1-14. doi: 10.1016/j.ceca.2018.12.006.
9. Fiedler, L.R., Chapman, K., Xie, M., Maifoshie, E., Jenkins, M., Golforoush, P.A., et al. (2019). MAP4K4 Inhibition Promotes Survival of Human Stem Cell-Derived Cardiomyocytes and Reduces Infarct Size In Vivo. Cell Stem Cell 24(4), 579-591.e512. doi: 10.1016/j.stem.2019.01.013.
10. Geraets, I.M.E., Chanda, D., van Tienen, F.H.J., van den Wijngaard, A., Kamps, R., Neumann, D., et al. (2018). Human embryonic stem cell-derived cardiomyocytes as an in vitro model to study cardiac insulin resistance. Biochim Biophys Acta Mol Basis Dis 1864(5 Pt B), 1960-1967. doi: 10.1016/j.bbadis.2017.12.025.
11. Graneli, C., Hicks, R., Brolen, G., Synnergren, J., and Sartipy, P. (2019). Diabetic Cardiomyopathy Modelling Using Induced Pluripotent Stem Cell Derived Cardiomyocytes: Recent Advances and Emerging Models. Stem Cell Rev Rep 15(1), 13-22. doi: 10.1007/s12015-018-9858-1.
12. Hidalgo, A., Glass, N., Ovchinnikov, D., Yang, S.K., Zhang, X., Mazzone, S., et al. (2018). Modelling ischemia-reperfusion injury (IRI) in vitro using metabolically matured induced pluripotent stem cell-derived cardiomyocytes. APL Bioeng 2(2), 026102. doi: 10.1063/1.5000746.
13. Hoes, M.F., Tromp, J., Ouwerkerk, W., Bomer, N., Oberdorf-Maass, S.U., Samani, N.J., et al. (2019). The role of cathepsin D in the pathophysiology of heart failure and its potentially beneficial properties: a translational approach. Eur J Heart Fail. doi: 10.1002/ejhf.1674.
14. Kirby, R.J., Divlianska, D.B., Whig, K., Bryan, N., Morfa, C.J., Koo, A., et al. (2018). Discovery of Novel Small-Molecule Inducers of Heme Oxygenase-1 That Protect Human iPSC-Derived Cardiomyocytes from Oxidative Stress. J Pharmacol Exp Ther 364(1), 87-96. doi: 10.1124/jpet.117.243717.
15. Kumar, A., Thomas, S.K., Wong, K.C., Lo Sardo, V., Cheah, D.S., Hou, Y.H., et al. (2019). Mechanical activation of noncoding-RNA-mediated regulation of disease-associated

phenotypes in human cardiomyocytes. Nat Biomed Eng 3(2), 137-146. doi: 10.1038/s41551-018-0344-5.

16. Lemme, M., Braren, I., Prondzynski, M., Aksehirlioglu, B., Ulmer, B.M., Schulze, M.L., et al. (2019). Chronic intermittent tachypacing by an optogenetic approach induces arrhythmia vulnerability in human engineered heart tissue. Cardiovasc Res. doi: 10.1093/cvr/cvz245.
17. Lu, Y., Bu, M., and Yun, H. (2019). Sevoflurane prevents hypoxia/reoxygenation-induced cardiomyocyte apoptosis by inhibiting PI3KC3-mediated autophagy. Hum Cell 32(2), 150-159. doi: 10.1007/s13577-018-00230-4.
18. Martewicz, S., Gabrel, G., Campesan, M., Canton, M., Di Lisa, F., and Elvassore, N. (2018). Live Cell Imaging in Microfluidic Device Proves Resistance to Oxygen/Glucose Deprivation in Human Induced Pluripotent Stem Cell-Derived Cardiomyocytes. Anal Chem 90(9), 5687-5695. doi: 10.1021/acs.analchem.7b05347.
19. Mirtschink, P., Bischof, C., Pham, M.D., Sharma, R., Khadayate, S., Rossi, G., et al. (2019). Inhibition of the Hypoxia-Inducible Factor 1alpha-Induced Cardiospecific HERNA1 Enhance-Templated RNA Protects From Heart Disease. Circulation 139(24), 2778-2792. doi: 10.1161/circulationaha.118.036769.
20. Mo, B., Wu, X., Wang, X., Xie, J., Ye, Z., and Li, L. (2019). miR-30e-5p Mitigates Hypoxia-Induced Apoptosis in Human Stem Cell-Derived Cardiomyocytes by Suppressing Bim. Int J Biol Sci 15(5), 1042-1051. doi: 10.7150/ijbs.31099.
21. Naftali-Shani, N., Molotski, N., Nevo-Caspi, Y., Arad, M., Kuperstein, R., Amit, U., et al. (2018). Modeling Peripartum Cardiomyopathy With Human Induced Pluripotent Stem Cells Reveals Distinctive Abnormal Function of Cardiomyocytes. Circulation 138(23), 2721-2723. doi: 10.1161/circulationaha.118.035950.
22. Ng, K.M., Lau, Y.M., Dhandhania, V., Cai, Z.J., Lee, Y.K., Lai, W.H., et al. (2018). Empagliflozin Ammeliorates High Glucose Induced-Cardiac Dysfuntion in Human iPSC-Derived Cardiomyocytes. Sci Rep 8(1), 14872. doi: 10.1038/s41598-018-33293-2.
23. Pant, T., Mishra, M.K., Bai, X., Ge, Z.D., Bosnjak, Z.J., and Dhanasekaran, A. (2019). Microarray analysis of long non-coding RNA and mRNA expression profiles in diabetic cardiomyopathy using human induced pluripotent stem cell-derived cardiomyocytes. Diab Vasc Dis Res 16(1), 57-68. doi: 10.1177/1479164118813888.
24. Rampoldi, A., Singh, M., Wu, Q., Duan, M., Jha, R., Maxwell, J.T., et al. (2019). Cardiac Toxicity From Ethanol Exposure in Human-Induced Pluripotent Stem Cell-Derived Cardiomyocytes. Toxicol Sci 169(1), 280-292. doi: 10.1093/toxsci/kfz038.
25. Ronaldson-Bouchard, K., Ma, S.P., Yeager, K., Chen, T., Song, L., Sirabella, D., et al. (2018). Advanced maturation of human cardiac tissue grown from pluripotent stem cells. Nature 556(7700), 239-243. doi: 10.1038/s41586-018-0016-3.
26. Rosales, W., and Lizcano, F. (2018). The Histone Demethylase JMJD2A Modulates the Induction of Hypertrophy Markers in iPSC-Derived Cardiomyocytes. Front Genet 9, 14. doi: 10.3389/fgene.2018.00014.
27. Sass, G., Madigan, R.T., Joubert, L.M., Bozzi, A., Sayed, N., Wu, J.C., et al. (2019a). A Combination of Itraconazole and Amiodarone Is Highly Effective against Trypanosoma cruzi Infection of Human Stem Cell-Derived Cardiomyocytes. Am J Trop Med Hyg 101(2), 383-391. doi: 10.4269/ajtmh.19-0023.
28. Sass, G., Tsamo, A.T., Chounda, G.A.M., Nangmo, P.K., Sayed, N., Bozzi, A., et al. (2019b).

Vismione B Interferes with Trypanosoma cruzi Infection of Vero Cells and Human Stem Cell-Derived Cardiomyocytes. Am J Trop Med Hyg 101(6), 1359-1368. doi: 10.4269/ajtmh.19-0350.
29. Scrimgeour, N.R., Wrobel, A., Pinho, M.J., and Hoydal, M.A. (2019). microRNA-451a prevents activation of matrix metalloproteinases 2/9 in human cardiomyocytes during pathological stress stimulation. Am J Physiol Cell Physiol. doi: 10.1152/ajpcell.00204.2019.
30. Sebastiao, M.J., Gomes-Alves, P., Reis, I., Sanchez, B., Palacios, I., Serra, M., et al. (2020). Bioreactor-based 3D human myocardial ischemia/reperfusion in vitro model: a novel tool to unveil key paracrine factors upon acute myocardial infarction. Transl Res 215, 57-74. doi: 10.1016/j.trsl.2019.09.001.
31. Sebastiao, M.J., Serra, M., Pereira, R., Palacios, I., Gomes-Alves, P., and Alves, P.M. (2019). Human cardiac progenitor cell activation and regeneration mechanisms: exploring a novel myocardial ischemia/reperfusion in vitro model. Stem Cell Res Ther 10(1), 77. doi: 10.1186/s13287-019-1174-4.
32. Sewanan, L.R., Schwan, J., Kluger, J., Park, J., Jacoby, D.L., Qyang, Y., et al. (2019). Extracellular Matrix From Hypertrophic Myocardium Provokes Impaired Twitch Dynamics in Healthy Cardiomyocytes. JACC Basic Transl Sci 4(4), 495-505. doi: 10.1016/j.jacbts.2019.03.004.
33. Turnbull, I.C., Mayourian, J., Murphy, J.F., Stillitano, F., Ceholski, D.K., and Costa, K.D. (2018). Cardiac Tissue Engineering Models of Inherited and Acquired Cardiomyopathies. Methods Mol Biol 1816, 145-159. doi: 10.1007/978-1-4939-8597-5_11.
34. Ward, M.C., and Gilad, Y. (2019). A generally conserved response to hypoxia in iPSC-derived cardiomyocytes from humans and chimpanzees. Elife 8. doi: 10.7554/eLife.42374.
35. Wnorowski, A., Sharma, A., Chen, H., Wu, H., Shao, N.Y., Sayed, N., et al. (2019). Effects of Spaceflight on Human Induced Pluripotent Stem Cell-Derived Cardiomyocyte Structure and Function. Stem Cell Reports 13(6), 960-969. doi: 10.1016/j.stemcr.2019.10.006.
36. Wu, D., Zhang, Q., Yu, Y., Zhang, Y., Zhang, M., Liu, Q., et al. (2018). Oleanolic Acid, a Novel Endothelin A Receptor Antagonist, Alleviated High Glucose-Induced Cardiomyocytes Injury. Am J Chin Med 46(6), 1187-1201. doi: 10.1142/s0192415x18500623.
37. Zhang, M.Y., Guo, F.F., Wu, H.W., Yu, Y.Y., Wei, J.Y., Wang, S.F., et al. (2017). DanHong injection targets endothelin receptor type B and angiotensin II receptor type 1 in protection against cardiac hypertrophy. Oncotarget 8(61), 103393-103409. doi: 10.18632/oncotarget.21900.
38. Zhao, X., Chen, H., Xiao, D., Yang, H., Itzhaki, I., Qin, X., et al. (2018). Comparison of Non-human Primate versus Human Induced Pluripotent Stem Cell-Derived Cardiomyocytes for Treatment of Myocardial Infarction. Stem Cell Reports 10(2), 422-435. doi: 10.1016/j.stemcr.2018.01.002.

References for Figure 2B

Maladaptive Hypertrophy models

1. Aggarwal, P., Turner, A., Matter, A., Kattman, S.J., Stoddard, A., Lorier, R., et al. (2014). RNA expression profiling of human iPSC-derived cardiomyocytes in a cardiac hypertrophy model. PLoS One 9(9), e108051. doi: 10.1371/journal.pone.0108051.
2. Carlson, C., Koonce, C., Aoyama, N., Einhorn, S., Fiene, S., Thompson, A., et al. (2013). Phenotypic screening with human iPS cell-derived cardiomyocytes: HTS-compatible assays for interrogating cardiac hypertrophy. J Biomol Screen 18(10), 1203-1211. doi: 10.1177/1087057113500812.
3. Cui, H., Schlesinger, J., Schoenhals, S., Tonjes, M., Dunkel, I., Meierhofer, D., et al. (2016). Phosphorylation of the chromatin remodeling factor DPF3a induces cardiac hypertrophy through releasing HEY repressors from DNA. Nucleic Acids Res 44(6), 2538-2553. doi: 10.1093/nar/gkv1244.
4. Foldes, G., Matsa, E., Kriston-Vizi, J., Leja, T., Amisten, S., Kolker, L., et al. (2014). Aberrant alpha-adrenergic hypertrophic response in cardiomyocytes from human induced pluripotent cells. Stem Cell Reports 3(5), 905-914. doi: 10.1016/j.stemcr.2014.09.002.
5. Foldes, G., Mioulane, M., Wright, J.S., Liu, A.Q., Novak, P., Merkely, B., et al. (2011). Modulation of human embryonic stem cell-derived cardiomyocyte growth: a testbed for studying human cardiac hypertrophy? J Mol Cell Cardiol 50(2), 367-376. doi: 10.1016/j.yjmcc.2010.10.029.
6. Gao, G., Xie, A., Huang, S.-C., Zhou, A., Zhang, J., Herman, A.M., et al. (2011). Role of RBM25/LUC7L3 in abnormal cardiac sodium channel splicing regulation in human heart failure. Circulation 124(10), 1124-1131. doi: 10.1161/CIRCULATIONAHA.111.044495.
7. Gao, G., Xie, A., Zhang, J., Herman, A.M., Jeong, E.M., Gu, L., et al. (2013). Unfolded protein response regulates cardiac sodium current in systolic human heart failure. Circ Arrhythm Electrophysiol 6(5), 1018-1024. doi: 10.1161/circep.113.000274.
8. Gesmundo, I., Miragoli, M., Carullo, P., Trovato, L., Larcher, V., Di Pasquale, E., et al. (2017). Growth hormone-releasing hormone attenuates cardiac hypertrophy and improves heart function in pressure overload-induced heart failure. Proc Natl Acad Sci U S A 114(45), 12033-12038. doi: 10.1073/pnas.1712612114.
9. Hoes, M.F., Tromp, J., Ouwerkerk, W., Bomer, N., Oberdorf-Maass, S.U., Samani, N.J., et al. (2019). The role of cathepsin D in the pathophysiology of heart failure and its potentially beneficial properties: a translational approach. Eur J Heart Fail. doi: 10.1002/ejhf.1674.
10. Martin, T.P., Hortigon-Vinagre, M.P., Findlay, J.E., Elliott, C., Currie, S., and Baillie, G.S. (2014). Targeted disruption of the heat shock protein 20-phosphodiesterase 4D (PDE4D) interaction protects against pathological cardiac remodelling in a mouse model of hypertrophy. FEBS Open Bio 4, 923-927. doi: 10.1016/j.fob.2014.10.011.
11. Mathieu, S., El Khoury, N., Rivard, K., Gelinas, R., Goyette, P., Paradis, P., et al. (2016). Reduction in Na(+) current by angiotensin II is mediated by PKCalpha in mouse and human-induced pluripotent stem cell-derived cardiomyocytes. Heart Rhythm 13(6), 1346-1354. doi: 10.1016/j.hrthm.2016.02.015.
12. Mirtschink, P., Bischof, C., Pham, M.D., Sharma, R., Khadayate, S., Rossi, G., et al. (2019). Inhibition of the Hypoxia-Inducible Factor 1alpha-Induced Cardiospecific HERNA1 Enhance-Templated RNA Protects From Heart Disease. Circulation 139(24), 2778-2792. doi: 10.1161/circulationaha.118.036769.

13. Nagai, H., Satomi, T., Abiru, A., Miyamoto, K., Nagasawa, K., Maruyama, M., et al. (2017). Antihypertrophic Effects of Small Molecules that Maintain Mitochondrial ATP Levels Under Hypoxia. EBioMedicine 24, 147-158. doi: 10.1016/j.ebiom.2017.09.022.
14. Qin, P., Arabacilar, P., Bernard, R.E., Bao, W., Olzinski, A.R., Guo, Y., et al. (2017). Activation of the Amino Acid Response Pathway Blunts the Effects of Cardiac Stress. J Am Heart Assoc 6(5). doi: 10.1161/jaha.116.004453.
15. Ronaldson-Bouchard, K., Ma, S.P., Yeager, K., Chen, T., Song, L., Sirabella, D., et al. (2018). Advanced maturation of human cardiac tissue grown from pluripotent stem cells. Nature 556(7700), 239-243. doi: 10.1038/s41586-018-0016-3.
16. Rosales, W., and Lizcano, F. (2018). The Histone Demethylase JMJD2A Modulates the Induction of Hypertrophy Markers in iPSC-Derived Cardiomyocytes. Front Genet 9, 14. doi: 10.3389/fgene.2018.00014.
17. Rupert, C.E., Chang, H.H., and Coulombe, K.L. (2017). Hypertrophy changes 3D shape of hiPSC-cardiomyocytes: Implications for cellular maturation in regenerative medicine. Cell Mol Bioeng 10(1), 54-62. doi: 10.1007/s12195-016-0462-7.
18. Scrimgeour, N.R., Wrobel, A., Pinho, M.J., and Hoydal, M.A. (2019). microRNA-451a prevents activation of matrix metalloproteinases 2/9 in human cardiomyocytes during pathological stress stimulation. Am J Physiol Cell Physiol. doi: 10.1152/ajpcell.00204.2019.
19. Sewanan, L.R., Schwan, J., Kluger, J., Park, J., Jacoby, D.L., Qyang, Y., et al. (2019). Extracellular Matrix From Hypertrophic Myocardium Provokes Impaired Twitch Dynamics in Healthy Cardiomyocytes. JACC Basic Transl Sci 4(4), 495-505. doi: 10.1016/j.jacbts.2019.03.004.
20. Tanaka, A., Yuasa, S., Mearini, G., Egashira, T., Seki, T., Kodaira, M., et al. (2014). Endothelin-1 induces myofibrillar disarray and contractile vector variability in hypertrophic cardiomyopathy-induced pluripotent stem cell-derived cardiomyocytes. J Am Heart Assoc 3(6), e001263. doi: 10.1161/jaha.114.001263.
21. Tiburcy, M., Hudson, J.E., Balfanz, P., Schlick, S., Meyer, T., Chang Liao, M.L., et al. (2017). Defined Engineered Human Myocardium With Advanced Maturation for Applications in Heart Failure Modeling and Repair. Circulation 135(19), 1832-1847. doi: 10.1161/circulationaha.116.024145.
22. Wang, Z., Zhang, X.J., Ji, Y.X., Zhang, P., Deng, K.Q., Gong, J., et al. (2016). The long noncoding RNA Chaer defines an epigenetic checkpoint in cardiac hypertrophy. Nat Med 22(10), 1131-1139. doi: 10.1038/nm.4179.
23. Zhang, M.Y., Guo, F.F., Wu, H.W., Yu, Y.Y., Wei, J.Y., Wang, S.F., et al. (2017). DanHong injection targets endothelin receptor type B and angiotensin II receptor type 1 in protection against cardiac hypertrophy. Oncotarget 8(61), 103393-103409. doi: 10.18632/oncotarget.21900.

Ischemia/Reperfusion models

1. Chen, T., and Vunjak-Novakovic, G. (2019). Human Tissue-Engineered Model of Myocardial Ischemia-Reperfusion Injury. Tissue Eng Part A 25(9-10), 711-724. doi: 10.1089/ten.TEA.2018.0212.
2. Fernandez-Morales, J.C., Hua, W., Yao, Y., and Morad, M. (2019). Regulation of Ca(2+) signaling by acute hypoxia and acidosis in cardiomyocytes derived from human induced pluripotent stem cells. Cell Calcium 78, 1-14. doi: 10.1016/j.ceca.2018.12.006.
3. Fiedler, L.R., Chapman, K., Xie, M., Maifoshie, E., Jenkins, M., Golforoush, P.A., et al. (2019).

MAP4K4 Inhibition Promotes Survival of Human Stem Cell-Derived Cardiomyocytes and Reduces Infarct Size In Vivo. Cell Stem Cell 24(4), 579-591.e512. doi: 10.1016/j.stem.2019.01.013.
4. Hidalgo, A., Glass, N., Ovchinnikov, D., Yang, S.K., Zhang, X., Mazzone, S., et al. (2018). Modelling ischemia-reperfusion injury (IRI) in vitro using metabolically matured induced pluripotent stem cell-derived cardiomyocytes. APL Bioeng 2(2), 026102. doi: 10.1063/1.5000746.
5. Hsieh, A., Feric, N.T., and Radisic, M. (2015). Combined hypoxia and sodium nitrite pretreatment for cardiomyocyte protection in vitro. Biotechnol Prog 31(2), 482-492. doi: 10.1002/btpr.2039.
6. Kirby, R.J., Divlianska, D.B., Whig, K., Bryan, N., Morfa, C.J., Koo, A., et al. (2018). Discovery of Novel Small-Molecule Inducers of Heme Oxygenase-1 That Protect Human iPSC-Derived Cardiomyocytes from Oxidative Stress. J Pharmacol Exp Ther 364(1), 87-96. doi: 10.1124/jpet.117.243717.
7. Lu, Y., Bu, M., and Yun, H. (2019). Sevoflurane prevents hypoxia/reoxygenation-induced cardiomyocyte apoptosis by inhibiting PI3KC3-mediated autophagy. Hum Cell 32(2), 150-159. doi: 10.1007/s13577-018-00230-4.
8. Martewicz, S., Gabrel, G., Campesan, M., Canton, M., Di Lisa, F., and Elvassore, N. (2018). Live Cell Imaging in Microfluidic Device Proves Resistance to Oxygen/Glucose Deprivation in Human Induced Pluripotent Stem Cell-Derived Cardiomyocytes. Anal Chem 90(9), 5687-5695. doi: 10.1021/acs.analchem.7b05347.
9. Mo, B., Wu, X., Wang, X., Xie, J., Ye, Z., and Li, L. (2019). miR-30e-5p Mitigates Hypoxia-Induced Apoptosis in Human Stem Cell-Derived Cardiomyocytes by Suppressing Bim. Int J Biol Sci 15(5), 1042-1051. doi: 10.7150/ijbs.31099.
10. Sebastiao, M.J., Gomes-Alves, P., Reis, I., Sanchez, B., Palacios, I., Serra, M., et al. (2020). Bioreactor-based 3D human myocardial ischemia/reperfusion in vitro model: a novel tool to unveil key paracrine factors upon acute myocardial infarction. Transl Res 215, 57-74. doi: 10.1016/j.trsl.2019.09.001.
11. Sebastiao, M.J., Serra, M., Pereira, R., Palacios, I., Gomes-Alves, P., and Alves, P.M. (2019). Human cardiac progenitor cell activation and regeneration mechanisms: exploring a novel myocardial ischemia/reperfusion in vitro model. Stem Cell Res Ther 10(1), 77. doi: 10.1186/s13287-019-1174-4.
12. Ward, M.C., and Gilad, Y. (2019). A generally conserved response to hypoxia in iPSC-derived cardiomyocytes from humans and chimpanzees. Elife 8. doi: 10.7554/eLife.42374.
13. Wei, H., Wang, C., Guo, R., Takahashi, K., and Naruse, K. (2019). Development of a model of ischemic heart disease using cardiomyocytes differentiated from human induced pluripotent stem cells. Biochem Biophys Res Commun 520(3), 600-605. doi: 10.1016/j.bbrc.2019.09.119.
14. Zhao, X., Chen, H., Xiao, D., Yang, H., Itzhaki, I., Qin, X., et al. (2018). Comparison of Non-human Primate versus Human Induced Pluripotent Stem Cell-Derived Cardiomyocytes for Treatment of Myocardial Infarction. Stem Cell Reports 10(2), 422-435. doi: 10.1016/j.stemcr.2018.01.002.

Diabetes models

1. Canfield, S.G., Sepac, A., Sedlic, F., Muravyeva, M.Y., Bai, X., and Bosnjak, Z.J. (2012). Marked

hyperglycemia attenuates anesthetic preconditioning in human-induced pluripotent stem cell-derived cardiomyocytes. Anesthesiology 117(4), 735-744. doi: 10.1097/ALN.0b013e3182655e96.

2. Canfield, S.G., Zaja, I., Godshaw, B., Twaroski, D., Bai, X., and Bosnjak, Z.J. (2016). High Glucose Attenuates Anesthetic Cardioprotection in Stem-Cell-Derived Cardiomyocytes: The Role of Reactive Oxygen Species and Mitochondrial Fission. Anesth Analg 122(5), 1269-1279. doi: 10.1213/ane.0000000000001254.
3. Chanda, D., Oligschlaeger, Y., Geraets, I., Liu, Y., Zhu, X., Li, J., et al. (2017). 2-Arachidonoylglycerol ameliorates inflammatory stress-induced insulin resistance in cardiomyocytes. Journal of Biological Chemistry 292(17), 7105-7114. doi: 10.1074/jbc.M116.767384.
4. Drawnel, F.M., Boccardo, S., Prummer, M., Delobel, F., Graff, A., Weber, M., et al. (2014). Disease modeling and phenotypic drug screening for diabetic cardiomyopathy using human induced pluripotent stem cells. Cell Rep 9(3), 810-821. doi: 10.1016/j.celrep.2014.09.055.
5. Geraets, I.M.E., Chanda, D., van Tienen, F.H.J., van den Wijngaard, A., Kamps, R., Neumann, D., et al. (2018). Human embryonic stem cell-derived cardiomyocytes as an in vitro model to study cardiac insulin resistance. Biochim Biophys Acta Mol Basis Dis 1864(5 Pt B), 1960-1967. doi: 10.1016/j.bbadis.2017.12.025.
6. Graneli, C., Hicks, R., Brolen, G., Synnergren, J., and Sartipy, P. (2019). Diabetic Cardiomyopathy Modelling Using Induced Pluripotent Stem Cell Derived Cardiomyocytes: Recent Advances and Emerging Models. Stem Cell Rev Rep 15(1), 13-22. doi: 10.1007/s12015-018-9858-1.
7. Kikuchi, C., Bienengraeber, M., Canfield, S., Koopmeiner, A., Schafer, R., Bosnjak, Z.J., et al. (2015). Comparison of Cardiomyocyte Differentiation Potential Between Type 1 Diabetic Donor- and Nondiabetic Donor-Derived Induced Pluripotent Stem Cells. Cell Transplant 24(12), 2491-2504. doi: 10.3727/096368914x685762.
8. Liu, Y., Steinbusch, L.K.M., Nabben, M., Kapsokalyvas, D., van Zandvoort, M., Schönleitner, P., et al. (2017). Palmitate-Induced Vacuolar-Type H⁺-ATPase Inhibition Feeds Forward Into Insulin Resistance and Contractile Dysfunction. Diabetes 66(6), 1521. doi: 10.2337/db16-0727.
9. Ng, K.M., Lau, Y.M., Dhandhania, V., Cai, Z.J., Lee, Y.K., Lai, W.H., et al. (2018). Empagliflozin Ammeliorates High Glucose Induced-Cardiac Dysfuntion in Human iPSC-Derived Cardiomyocytes. Sci Rep 8(1), 14872. doi: 10.1038/s41598-018-33293-2.
10. Pant, T., Mishra, M.K., Bai, X., Ge, Z.D., Bosnjak, Z.J., and Dhanasekaran, A. (2019). Microarray analysis of long non-coding RNA and mRNA expression profiles in diabetic cardiomyopathy using human induced pluripotent stem cell-derived cardiomyocytes. Diab Vasc Dis Res 16(1), 57-68. doi: 10.1177/1479164118813888.
11. Sepac, A., Sedlic, F., Si-Tayeb, K., Lough, J., Duncan, S.A., Bienengraeber, M., et al. (2010). Isoflurane preconditioning elicits competent endogenous mechanisms of protection from oxidative stress in cardiomyocytes derived from human embryonic stem cells. Anesthesiology 113(4), 906-916. doi: 10.1097/ALN.0b013e3181eff6b7.
12. Wu, D., Zhang, Q., Yu, Y., Zhang, Y., Zhang, M., Liu, Q., et al. (2018). Oleanolic Acid, a Novel Endothelin A Receptor Antagonist, Alleviated High Glucose-Induced Cardiomyocytes Injury. Am J Chin Med 46(6), 1187-1201. doi: 10.1142/s0192415x18500623.

Infectious disease models

1. Bozzi, A., Sayed, N., Matsa, E., Sass, G., Neofytou, E., Clemons, K.V., et al. (2019). Using Human Induced Pluripotent Stem Cell-Derived Cardiomyocytes as a Model to Study Trypanosoma cruzi Infection. Stem Cell Reports 12(6), 1232-1241. doi: 10.1016/j.stemcr.2019.04.017.
2. da Silva Lara, L., Andrade-Lima, L., Magalhaes Calvet, C., Borsoi, J., Lopes Alberto Duque, T., Henriques-Pons, A., et al. (2018). Trypanosoma cruzi infection of human induced pluripotent stem cell-derived cardiomyocytes: an in vitro model for drug screening for Chagas disease. Microbes Infect 20(5), 312-316. doi: 10.1016/j.micinf.2018.03.002.
3. Sass, G., Madigan, R.T., Joubert, L.M., Bozzi, A., Sayed, N., Wu, J.C., et al. (2019a). A Combination of Itraconazole and Amiodarone Is Highly Effective against Trypanosoma cruzi Infection of Human Stem Cell-Derived Cardiomyocytes. Am J Trop Med Hyg 101(2), 383-391. doi: 10.4269/ajtmh.19-0023.
4. Sass, G., Tsamo, A.T., Chounda, G.A.M., Nangmo, P.K., Sayed, N., Bozzi, A., et al. (2019b). Vismione B Interferes with Trypanosoma cruzi Infection of Vero Cells and Human Stem Cell-Derived Cardiomyocytes. Am J Trop Med Hyg 101(6), 1359-1368. doi: 10.4269/ajtmh.19-0350.
5. Scassa, M.E., Jaquenod de Giusti, C., Questa, M., Pretre, G., Richardson, G.A., Bluguermann, C., et al. (2011). Human embryonic stem cells and derived contractile embryoid bodies are susceptible to Coxsakievirus B infection and respond to interferon Ibeta treatment. Stem Cell Res 6(1), 13-22. doi: 10.1016/j.scr.2010.09.002.
6. Sharma, A., Marceau, C., Hamaguchi, R., Burridge, P.W., Rajarajan, K., Churko, J.M., et al. (2014). Human induced pluripotent stem cell-derived cardiomyocytes as an in vitro model for coxsackievirus B3-induced myocarditis and antiviral drug screening platform. Circ Res 115(6), 556-566. doi: 10.1161/circresaha.115.303810.
7. Yucel, G., Zhao, Z., El-Battrawy, I., Lan, H., Lang, S., Li, X., et al. (2017). Lipopolysaccharides induced inflammatory responses and electrophysiological dysfunctions in human-induced pluripotent stem cell derived cardiomyocytes. Sci Rep 7(1), 2935. doi: 10.1038/s41598-017-03147-4.

Spaceflight models

1. Acharya, A., Brungs, S., Lichterfeld, Y., Hescheler, J., Hemmersbach, R., Boeuf, H., et al. (2019). Parabolic, Flight-Induced, Acute Hypergravity and Microgravity Effects on the Beating Rate of Human Cardiomyocytes. Cells 8(4). doi: 10.3390/cells8040352.
2. Becker, B.V., Majewski, M., Abend, M., Palnek, A., Nestler, K., Port, M., et al. (2018a). Gene expression changes in human iPSC-derived cardiomyocytes after X-ray irradiation. Int J Radiat Biol 94(12), 1095-1103. doi: 10.1080/09553002.2018.1516908.
3. Becker, B.V., Seeger, T., Beiert, T., Antwerpen, M., Palnek, A., Port, M., et al. (2018b). Impact of Ionizing Radiation on Electrophysiological Behavior of Human-induced Ipsc-derived Cardiomyocytes on Multielectrode Arrays. Health Phys 115(1), 21-28. doi: 10.1097/hp.0000000000000817.
4. Wnorowski, A., Sharma, A., Chen, H., Wu, H., Shao, N.Y., Sayed, N., et al. (2019). Effects of Spaceflight on Human Induced Pluripotent Stem Cell-Derived Cardiomyocyte Structure and Function. Stem Cell Reports 13(6), 960-969. doi: 10.1016/j.stemcr.2019.10.006.

Fibrosis models

1. Kumar, A., Thomas, S.K., Wong, K.C., Lo Sardo, V., Cheah, D.S., Hou, Y.H., et al. (2019). Mechanical activation of noncoding-RNA-mediated regulation of disease-associated phenotypes in human cardiomyocytes. Nat Biomed Eng 3(2), 137-146. doi: 10.1038/s41551-018-0344-5.
2. Zhang, H., Tian, L., Shen, M., Tu, C., Wu, H., Gu, M., et al. (2019). Generation of Quiescent Cardiac Fibroblasts From Human Induced Pluripotent Stem Cells for In Vitro Modeling of Cardiac Fibrosis. Circ Res 125(5), 552-566. doi: 10.1161/circresaha.119.315491.

Other Pathology models

1. Acun, A., Nguyen, T.D., and Zorlutuna, P. (2019). In vitro aged, hiPSC-origin engineered heart tissue models with age-dependent functional deterioration to study myocardial infarction. Acta Biomater 94, 372-391. doi: 10.1016/j.actbio.2019.05.064.
2. Gaber, N., Gagliardi, M., Patel, P., Kinnear, C., Zhang, C., Chitayat, D., et al. (2013). Fetal reprogramming and senescence in hypoplastic left heart syndrome and in human pluripotent stem cells during cardiac differentiation. Am J Pathol 183(3), 720-734. doi: 10.1016/j.ajpath.2013.05.022.
3. Lemme, M., Braren, I., Prondzynski, M., Aksehirlioglu, B., Ulmer, B.M., Schulze, M.L., et al. (2019). Chronic intermittent tachypacing by an optogenetic approach induces arrhythmia vulnerability in human engineered heart tissue. Cardiovasc Res. doi: 10.1093/cvr/cvz245.
4. Naftali-Shani, N., Molotski, N., Nevo-Caspi, Y., Arad, M., Kuperstein, R., Amit, U., et al. (2018). Modeling Peripartum Cardiomyopathy With Human Induced Pluripotent Stem Cells Reveals Distinctive Abnormal Function of Cardiomyocytes. Circulation 138(23), 2721-2723. doi: 10.1161/circulationaha.118.035950.
5. Rampoldi, A., Singh, M., Wu, Q., Duan, M., Jha, R., Maxwell, J.T., et al. (2019). Cardiac Toxicity From Ethanol Exposure in Human-Induced Pluripotent Stem Cell-Derived Cardiomyocytes. Toxicol Sci 169(1), 280-292. doi: 10.1093/toxsci/kfz038.
6. Turnbull, I.C., Mayourian, J., Murphy, J.F., Stillitano, F., Ceholski, D.K., and Costa, K.D. (2018). Cardiac Tissue Engineering Models of Inherited and Acquired Cardiomyopathies. Methods Mol Biol 1816, 145-159. doi: 10.1007/978-1-4939-8597-5_11.
7. Voges, H.K., Mills, R.J., Elliott, D.A., Parton, R.G., Porrello, E.R., and Hudson, J.E. (2017). Development of a human cardiac organoid injury model reveals innate regenerative potential. Development 144(6), 1118. doi: 10.1242/dev.143966.
